# Dynamic Changes of Histone H1 Variants in Drug-Induced Senescence

**DOI:** 10.3390/genes17101274

**Published:** 2026-10-14

**Authors:** Nora Mittag, Adriana Birbio, Olga Delipetridou, Pau Homs-Aguadé, Carles Bonet-Costa, Albert Jordan

**Affiliations:** Molecular Biology Institute of Barcelona (IBMB-CSIC), 08028 Barcelona, Spain; noramittag@t-online.de (N.M.); adrianabirbio01@gmail.com (A.B.); olgadel21@gmail.com (O.D.); phabmc@ibmb.csic.es (P.H.-A.); cbobmc@ibmb.csic.es (C.B.-C.)

**Keywords:** linker histone, histone H1, histone variants, senescence, topoisomerase inhibitors, doxorubicin, breast cancer, chromatin, interferon-stimulated gene response, repetitive elements

## Abstract

**Background/Objectives:** Cellular senescence is a stress-induced state of irreversible cell cycle arrest triggered by diverse molecular stimuli. It is characterized by distinct phenotypic alterations, including the senescence-associated secretory phenotype (SASP), profound chromatin remodeling, and morphological changes. While senescent cells accumulate during physiological aging, they are also prevalent in pathological contexts such as cancer. Histone H1 is a crucial regulator of chromatin organization and gene expression, and emerging evidence suggests that its content and nuclear distribution change dynamically during senescence. **Methods:** Here, we investigated the expression and localization dynamics of six somatic H1 variants (H1.0, H1.2 to H1.5, and H1X) upon senescence induction in T47D breast cancer cells, evaluating their correlation with chromatin structural alterations, repetitive element expression, and interferon response activation. Senescence was triggered using doxorubicin, a topoisomerase inhibitor, among other drugs tested, which was confirmed by the accumulation of standard senescence markers (ß-galactosidase activity, *p*21^CIP1/WAF1^, H3K9me3, and γH2AX). **Results:** We observed variant-specific expression patterns, suggesting distinct functional contributions to senescence. Specifically, H1.0 was upregulated, whereas H1.3, H1.4 and H1.5 were downregulated. Furthermore, the nuclear redistribution of H1.2, H1.3, and H1.5 points to their active roles in local gene expression and silencing. These alterations coincided with the increased transcription of repetitive elements (LINEs, SINEs, LTRs and satellites) and the elevated expression of interferon-stimulated genes (*IFI27*, *OASL*, *IFIT3*), likely driven by localized chromatin decompression. Finally, functional knock-down experiments revealed that while the depletion of individual H1 variants yielded distinct cellular effects, it neither sufficed to fully mimic drug-induced senescence nor significantly altered the cellular response to senescence-inducing drugs. **Conclusions:** Taken together, our findings indicate that while H1 variant deregulation may be a hallmark of senescent breast cancer cells, and linker histones may actively modulate the threshold for senescence induction and genotoxic response, it represents one component of a broader, coordinated chromatin remodeling program.

## 1. Introduction

Senescence was initially described as a series of cellular alterations associated with aging. It is now more broadly defined as a signal transduction pathway that leads to an irreversible cell cycle arrest, accompanied by several characteristic phenotypic changes collectively known as the senescence-associated secretory phenotype (SASP) [1]. Its implementation primarily depends on the activation of the *p*21^CIP1/WAF1^, p53 and *p*16^INK4a^ tumor suppressor pathways. Beyond growth arrest, senescent cells undergo extensive metabolic reprogramming, structural and epigenetic changes affecting DNA transcription, protein expression and various other cellular processes [2,3]. Morphologically, senescent cells often display an enlarged, flattened structure [3] and an increased size of the nucleus [4]. Other common features are the development of senescence-associated heterochromatin foci (SAHF) and the formation of DNA damage foci [5]. Nevertheless, no single marker is universally reliable, and the accurate identification of senescence requires a combination of multiple measurements [4].

While senescence serves as a crucial tumor-suppressive mechanism by limiting cancer progression [6], the SASP can also activate the immune system leading to chronic inflammation and a pro-tumorigenic environment. Understanding the dual role of senescence in both disease progression and therapeutic response remains a key focus in biomedical research [7].

To study cellular processes of the senescence state, various drugs are used in research to generate this non-proliferation cell state. Doxorubicin and etoposide are widely used chemotherapeutics to induce senescence in different human cell lines [8,9,10,11]. Next to the DNA damage causing the topoisomerase inhibitors doxorubicin and etoposide, alisertib was also identified to induce senescence as an aurora kinase A inhibitor in several cell lines [12].

Upon the induction of cellular senescence, the gene expression profile changes drastically. Several studies analyzed the deregulation of various genomic elements and reported an upregulation of transposable element (TE) expression (e.g., ERV1, L1 or satellites) [11,13,14,15] and an immune response by the expression of interferon-stimulated genes (ISG) like *IFITM1*, *ISG15*, *IFIT*, *IFI27*, *IFIT2* and more [13,14]. Furthermore, a global loss of heterochromatin is described in senescent cells [13,16], leading to an increased level of ISG transcription [17].

The linker histone H1, as a key regulator of heterochromatin, could play an important role in the progression towards senescence. Even though histone H1 is highly conserved, the mammalian H1 family is evolutionarily diverse and includes up to eleven different isoforms, referred to as variants, of which seven can be found in human somatic cells: H1.0 to H1.5 and H1X [18]. Multiple studies presume that the H1 variants are not redundant and may have different specific functions (reviewed in Ref. [19]). The replication-dependent variants—basically, H1.2, H1.3, H1.4 and H1.5 (encoded by *HIST1H1B* to *HIST1H1E*)—are tightly coupled to active cell proliferation and canonical S-phase DNA synthesis. H1.0 (encoded by *H1F0*) is replication-independent and traditionally associated with terminally differentiated, non-dividing somatic cells. The least understood H1X (also named H1.10; encoded by *H1FX*) is also replication-independent and displays unique nuclear dynamics. Furthermore, they are suggested to not be randomly distributed within the nucleus, interacting with distinct regions of the genome [20]. More recent studies show more insights on the distribution within the genome and localization within the nucleus of the linker histone variants using chromatin immunoprecipitation assays with sequencing (ChIP-seq) and confocal immunofluorescence imaging using variant-specific antibodies [21,22,23]. The H1 distribution in T47D breast cancer cells was clustered into two groups. While H1.4 and H1X can be found in genomic regions with a high GC content distributed throughout the nuclei, H1.0, H1.2, H1.3 and H1.5 are more abundant in low-GC regions known to consist of low-activity chromatin and are enriched at the nuclear periphery [21,22]. Studies of H1 knock downs resulting in more opened and decompressed chromatin stress the involvement of H1 in stabilizing the structure of the genome [21,23,24]. It has been previously reported that H1 levels are reduced in senescent cells [25,26], although it remains elusive as to whether H1 deregulation is a cause for senescence induction or rather a consequence, and if all the variants behave redundantly.

The subject of this study is the role and dynamics of H1 and its variants in senescent cells. This research aims to investigate if H1 changes correlate with other alterations that occur upon senescence, such as shape modifications, heterochromatin dysregulation, derepression of repetitive elements, and the activation of interferon-stimulated genes, as well as if H1 alteration affects senescence induction. Taken together, our data suggests a model wherein therapy-induced senescence triggers a major remodeling of linker histone stoichiometry, dominated by H1.0 accumulation alongside the downregulation and spatial mislocalization of replication-dependent variants. This epigenetic transition may lead to localized heterochromatin erosion at repetitive loci, ultimately driving retrotransposon expression, cytosolic dsRNA accumulation, and ISG activation. These investigations can help to gain a better understanding of basic processes that are related to senescence, aging and diseases like cancer.

## 2. Materials and Methods

### 2.1. Cell Lines and Culturing Conditions

The human breast cancer cell line T47D was cultivated at 37 °C with 5% CO_2_ in RPMI 1640 medium supplemented with 10% fetal bovine serum, 2 mM L-glutamine, and 100 U/mL penicillin and streptomycin. Doxycycline (Dox)-inducible shRNA H1 knock-downs (KDs) were described in previous works [24,27,28]. Concretely, T47D H1.4sh [24], T47D H1.2sh [27] and T47D H1.0sh [27] cell lines were used to analyze single-H1 depletion. shRNA expression was induced with 6 days’ treatment of Dox, in which cells were passaged on day 3. Dox (Merck KGaA, Darmstat, Germany) was added at 2.5 µg/mL.

To generate a CRAMP1 knock-down, a shRNA cloned in pLKO.1 (Merck KGaA, Darmstat, Germany) was used (clone TRCN0000130478). Viral particles were obtained by transfecting HEK293T cells (Takara Bio USA, Mountain View, CA, USA), and infections were performed as reported before [29]. Infected cells were selected with 10 µg/mL puromycin (Merck KGaA, Darmstat, Germany) 24 h after infection.

### 2.2. Drug Treatments and SA-Β-Gal Staining

For senescence induction, three different drugs, each in three increasing concentrations, were used to treat T47D breast cancer cells for 48 h or 5 days. The treatment was followed by SA-β-gal staining to analyze the efficiency of the drug to induce senescence but not apoptosis. Therefore, the cells were stained with the senescence detection kit (Abcam, Cambridge, UK) for senescence-associated ß-galactosidase (SA-β-gal) staining. The staining was performed according to the manufacturer’s instructions. To store the cells, the staining was removed, and the cells were covered with 70% glycerol.

For replicable induction of senescence in T47D cells, the following treatment conditions were established: 0.05 μM doxorubicin (DR or DoxoR), 0.5 μM alisertib (AL) and 0.5 μM etoposide (ET) for 5 days. The media with drugs was renewed after 48 h of treatment.

### 2.3. RNA Extraction and Reverse Transcriptase (RT)-qPCR

Senescent cells were prepared by seeding 0.5 × 10^6^ cells in seven ∅ 10 cm dishes and treated with the drugs as described previously (dishes: 2× AL, 2× ET, 2× DR, 1× Control). To extract the RNA from the cells, the NZY Total RNA Isolation kit (NZYtech, Lisbon, Portugal) was used.

An amount of 100 ng of RNA of each sample and the SuperScript™ VILO™ cDNA Synthesis Kit (Thermo Fisher Scientific, Waltham, MA, USA) were used to generate the cDNA in the MJ Mini Bio-Rad thermal cycler (Bio-Rad Laboratories, Hercules, CA, USA). To analyze gene products, the PowerTrack^TM^ SYBR^TM^ Green MasterMix (Thermo Fisher Scientific, Waltham, MA, USA) and various specific oligonucleotides (sequence available at Supplementary Table S1) were used. Each value measured by the QuantStudio 5 machine (Thermo Fisher Scientific, Waltham, MA, USA) was corrected by the human housekeeping gene *GAPDH* and put in relation to the control.

### 2.4. Statistical Analysis of Data

Unpaired Student’s *t*-test was performed to determine significant differences between the control and the treated conditions. Where indicated, statistical significance was assessed using a one-way ANOVA followed by Dunnett’s multiple comparisons test as post hoc correction, with the untreated control group serving as the reference group. Furthermore, the standard deviation of the replicates was calculated and displayed in the graph as error bars.

For the analyses presented here, technical replicate measurements obtained from the same biological sample were treated as individual observations for statistical analysis in some cases (stated in the figure legends). Although this approach allowed for an exploratory assessment of differences between treatment conditions, these measurements do not represent independent biological replicates and therefore do not fully capture biological variability. Consequently, the statistical results, including the reported *p*-values, should be interpreted with caution and should be considered exploratory rather than definitive evidence of treatment effects. To circumvent this, additional independent experiments have been performed and are shown as Supplementary Material for comparison (Supplementary Figures S2, S4 and S5). This has allowed us to confirm and generalize the observed findings.

### 2.5. Histone Extraction, Gel-Electrophoresis and Western Blot

T47D cells were collected by trypsinization and centrifugation after the drug treatment. All the following steps were executed at 4 °C or on ice. The cell pellet was resuspended in 1 mL hypotonic solution (10 mM Tris–HCl (pH 8.0), 1 mM KCl, 1.5 mM MgCl_2_, 1 mM PMSF (phenylmethylsulfonyl fluoride), 1 mM DTT) and incubated for 30 min. After the centrifugation at 10,000× *g* for 10 min, the supernatant was discarded, the pellet was resuspended in 400 µL 0.2 M sulfuric acid and rotated for 30 min. The suspension was centrifuged at 16,000× *g* for 10 min. Then, 132 µL TCA (trichloroacetic acid) was added to the supernatant in order to precipitate histones during one hour of incubation. Subsequently, the solution was centrifuged at 16,000× *g* for 10 min and the pellet was carefully washed twice with pre-chilled acetone without resuspending. The pellet was centrifuged between and after the washing steps at 16,000× *g* for 10 min. After air-drying the pellet at room temperature, it was reconstituted in 100 µL nuclease-free water. To determine the protein concentration, the Micro BCA™ Protein Assay Kit (Thermo Fisher Scientific, Waltham, MA, USA) was used according to the manufacturer’s manual. Samples and standard dilution were measured in duplicate with a PowerWave XS spectrophotometer (BioTek, Winooski, VT, USA) at a wavelength of 562 nm.

For the protein separation, histone samples were exposed to 14% sodium dodecyl sulfate–polyacrylamide gel electrophoresis (SDS–PAGE). After running the gel, it was stained with Coomassie blue for one hour and destained overnight. The relative intensity of each H1 band was corrected by core histone H4 band.

To perform the Western blot after SDS–PAGE, proteins were transferred to a PVDF membrane and blocked with Odyssey blocking buffer (LI-COR Biosciences, Lincoln, NE, USA). The membrane was incubated with a specific primary antibody for the target of interest (H1.0–H1.5, H1X, H3K9me3, TOP2A) overnight at 4 °C. Subsequently, membrane was incubated with a secondary antibody conjugated to fluorescence (IRDye 680 goat anti-rabbit IgG or IRDye 800 goat anti-mouse IgG, LI-COR Biosciences, Lincoln, NE, USA) for one hour at room temperature. Bands were visualized with an Odyssey IR Imaging System (LI-COR Biosciences, Lincoln, NE, USA). The intensity of the band was measured with ImageJ using the gel analyzing tool and plotted with Excel.

### 2.6. Immunofluorescence Image Acquisition and Analysis

For the immunostaining, 20 × 10^3^ cells/well were seeded in the wells of a 24-well plate that contained a round cover slip in each well (thickness: 0.17 mm, 1.5 H, Marienfeld Superior, Paul Marienfeld, Lauda-Königshofen, Germany). Twelve wells were treated with DR while the other twelve wells were used as a control with media containing DMSO. The cells were fixated with 4% paraformaldehyde (20 min; RT), permeabilized by incubation with methanol (10 min, RT) and blocked in buffer (PBS, 5% BSA, 0.1% Triton) for one hour at RT. Cover slips were incubated with the primary antibody overnight at 4 °C and incubated with secondary antibody (Anti-Rabbit Fluor 488 and Anti-Mouse Fluor 568, Thermo Fisher Scientific, Waltham, MA, USA) for 1 h at RT in the dark. After washing with PBS-T, the cover slips were incubated with Hoechst staining for one hour at RT in the dark. Five washes with PBS-T and a final wash with water followed before the slips were mounted to a microscope slide with Pro-long glass (Thermo Fisher Scientific, Waltham, MA, USA). Slides were stored for 24–48 h in the dark at RT followed by storage at 4 °C until images were acquired. To visualize the abundance and location of two different molecules of interest within the cell on each slide, the seven antibody combinations shown in Supplementary Table S2 were used.

For the image acquisition, the Fusion software (v2.4.0.22) and a Dragonfly 505 multimodal spinning-disk confocal microscope (Andor Technologies, Belfast, UK) with a sCMOS Andor Sona 4.2B-11 camera and a ×100/NA-1.49 apochromat oil immersion objective were used. The fluorescing antibody conjugate Alexa-568 was excited by a 561 nm laser, Alexa-488 was excited by a 488 nm laser, and Hoechst was excited by a 405 nm laser. A ∅ 40 µm pinhole was used. The exposure time was adapted for each slide and altered from 5 to 180 ms to prevent pixel saturation but kept the same between the control and the treated sample. In the same way, the laser intensity was adapted. The magnification of the camera was set to ×2. Sixteen-bit pictures were taken along the *z*-axis in 0.11 µm intervals for a set range to create a confocal 3D image with a voxel size of 51 × 51 × 110 nm. The 3D image was deconvoluted (GPU-assisted deconvolution, Regularized Richardson-Lucy, 16 iterations) by the Fusion software.

The deconvoluted images were analyzed using ImageJ software (v1.54g). Brightness, contrast, and the minimum and maximum intensity values were adjusted to effectively eliminate background signal while preserving the fluorescence signal within the cell. Fluorescence signal intensity along a defined line was measured using the Plot Profile tool in ImageJ. The resulting data was subsequently plotted using Microsoft Excel. The mean signal intensity within the nucleus was measured with ImageJ by manually marking the nucleus area in 10 cells for each condition and antibody, and the mean of these 10 measurements was calculated and plotted with excel. Furthermore, the standard deviation of the mean intensity values was calculated and displayed in the graph as error bars. For the antibody J2, the signal within the whole image frame for only one cell was measured and depicted.

### 2.7. Primary Antibodies

Specific antibodies recognizing human H1 variants used for immunofluorescence or immunoblot were anti-H1.0/H5 clone 3H9 (Merck KGaA, Darmstat, Germany; 05–629-I), anti-H1.1 (Abcam, Cambridge, UK; ab279642), anti-H1.2 (Abcam, ab4086), anti-H1.3 (Abcam, ab203948), anti-H1.4 (Thermo Fisher Scientific, Waltham, MA, USA; 702876), anti-H1.5 (Thermo Fisher Scientific, Waltham, MA, USA; 711912) and anti-H1X (Abcam, ab31972). Other antibodies used in immunofluorescence and/or immunoblot were Lamin A (Abcam, ab8980), NPM1 (Abcam, ab10530), H3 (Abcam, ab203948), H3K9me3 (Abcam, ab8898), γH2AX (Abcam, ab3569), J2 recognizing dsRNA (Scicon, Redhill, UK; 10010200), and TOP2A (Santa Cruz Biotechnology, Dallas, TX, USA; sc-365916). All H1 variant antibodies were raised in rabbits, except for H1.0 antibody, which was raised in mice. Performance and specificity of H1 variant antibodies have been extensively validated in our previous publications [21,23].

## 3. Results

### 3.1. Upregulation of Senescence Markers upon Drug Treatment

In order to investigate histone H1 variants’ function and distribution, we have extensively used the breast cancer cell line T47D as a model before [21,22,23,24,27,28]. In order to test whether senescence could be induced in these cells, three drugs were tested: two topoisomerase inhibitors (doxorubicin and etoposide) and one aurora kinase inhibitor (alisertib). Drugs were used at different concentrations and treatment timings, and senescence was inferred by ß-galactosidase measurements (SA-ß-Gal staining) (Supplementary Figure S1). The drug concentrations and conditions that gave more senescence induction with reduced cell lethality were chosen: 0.05 μM doxorubicin (DR or DoxoR), 0.5 μM alisertib (AL) and 0.5 μM etoposide (ET) for 5 days.

**Figure 1:**
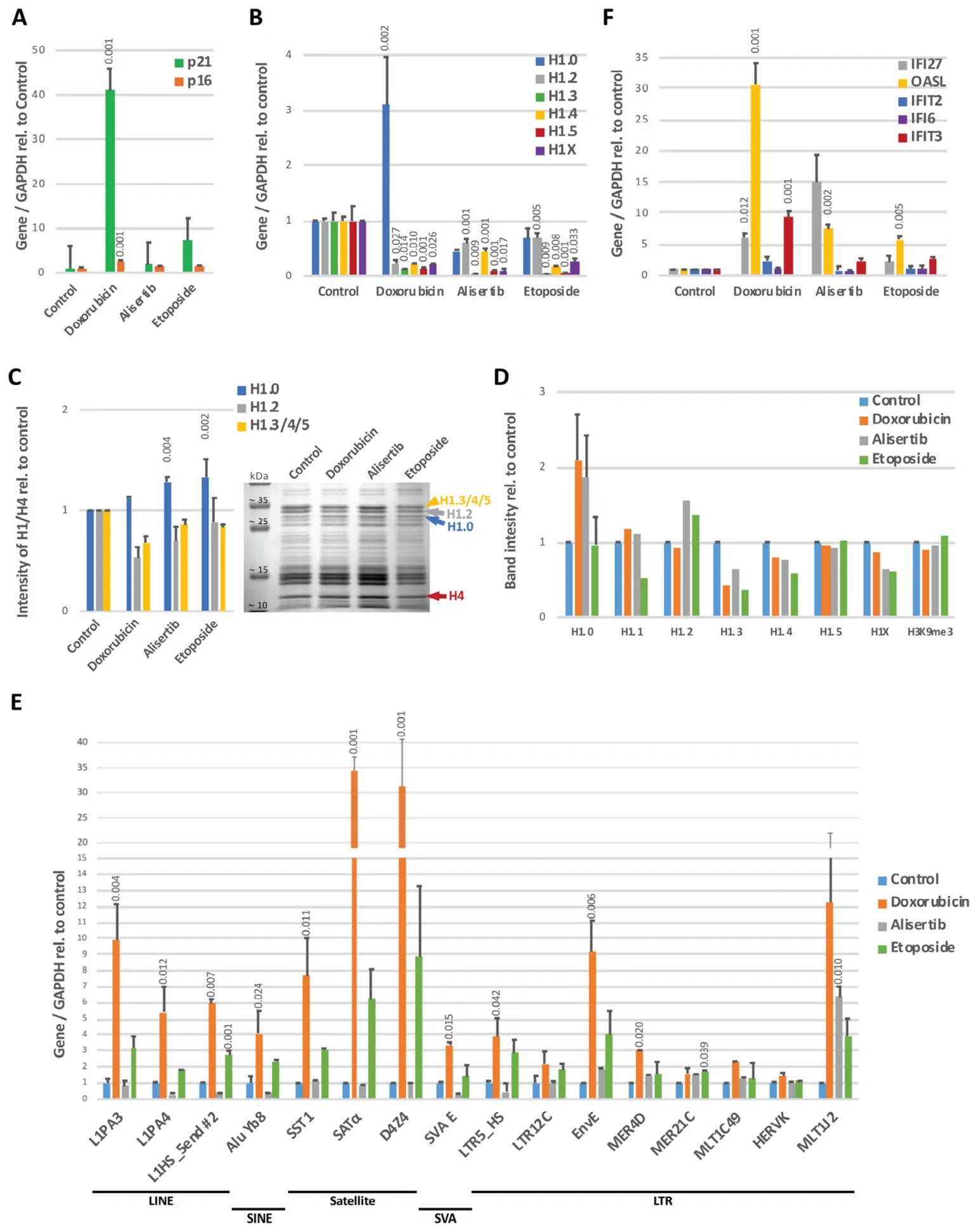
Effects of senescence induction in T47D cells on H1 variant expression and additional transcriptional consequences on senescence markers, TEs and ISGs. T47D cells were treated for five days with doxorubicin (DR), 0.05 µM; alisertib (AL), 0.5 µM; or etoposide (ET), 0.5 µM, or left untreated (control), and gene expression was measured by RT-qPCR with oligonucleotides specific for p21^CIP1/WAF1^ and p16^INK4a^ (**A**), six H1 variant genes (**B**), transposable elements of five classes (**E**), or five ISGs (**F**). GAPDH gene was used for normalization and data expressed relative to control. Statistical significance was assessed using a one-way ANOVA followed by Dunnett’s multiple comparisons test as post hoc correction, with the untreated control group serving as the reference group. *p*-value is depicted for significant changes (*p*-value < 0.05). A representative experiment analyzed in technical triplicates is shown. Additional representative experiments are shown in Supplementary Figure S2. (**C**) SDS–PAGE of histones extracted from T47D cells treated as before, as well as quantification of the Coomassie-stained bands corresponding to H1.0, H1.2, H1.3, H1.4, and H1.5, which were normalized to H4 core histone intensity and expressed relative to the untreated control. A representative SDS–PAGE is shown. Quantification corresponds to two independent experiments. Statistical significance was assessed using a one-way ANOVA followed by Dunnett’s multiple comparisons test as post hoc correction, with the untreated control group serving as the reference group. *p*-value is depicted for significant changes (*p*-value < 0.05). (**D**) Histone H1 variant expression upon drug treatment was measured by Western blot with variant-specific antibodies (see Supplementary Figure S3). Band intensities are shown for each H1 variant and H3K9me3 and expressed relative to the untreated control. For H1.0, the mean and standard deviation of the measurement on six membranes is depicted, as it was immunoblotted together with each of the other H1 variant antibodies. All graphs displaying error bars indicate the standard deviation of the replicates.

Additional markers of senescence are *p*16^INK4a^ and *p*21^CIP1/WAF1^ expression and the appearance of DNA damage-associated gamma-H2AX foci, in addition to cell and nuclei shape and size changes. In DoxoR-treated samples, a significant upregulation of senescence markers *p*21^CIP1/WAF1^ and *p*16^INK4a^ was observed (Figure 1A). Etoposide also induced *p*21^CIP1/WAF1^ to some extent, although statistical analysis of the alisertib and etoposide-treated sample did not indicate any significant changes in the expression of *p*16^INK4a^ and *p*21^CIP1/WAF1^. p53 was not analyzed, as T47D cells contain a missense mutation in the TP53 gene, resulting in non-functional p53.

**Figure 2:**
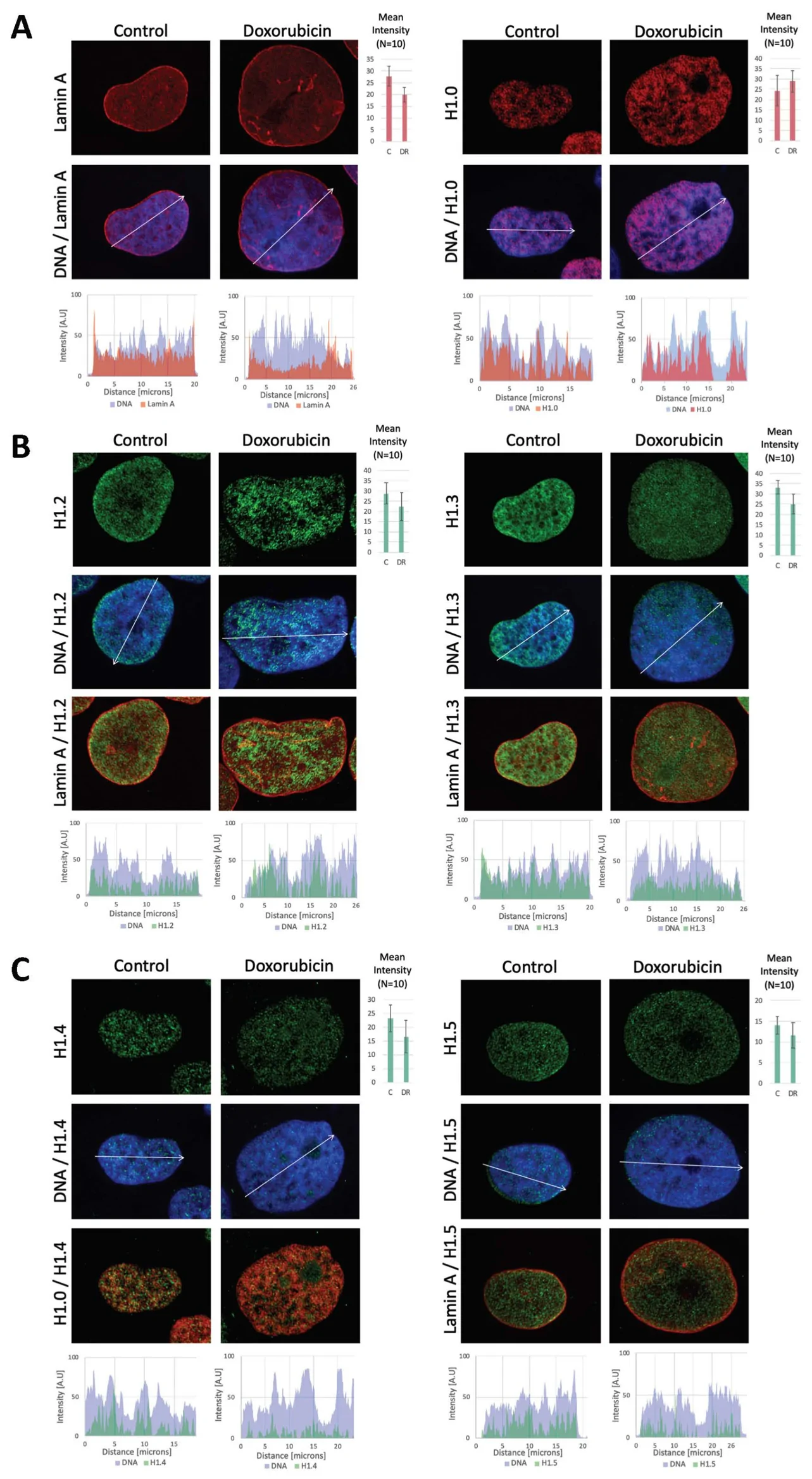

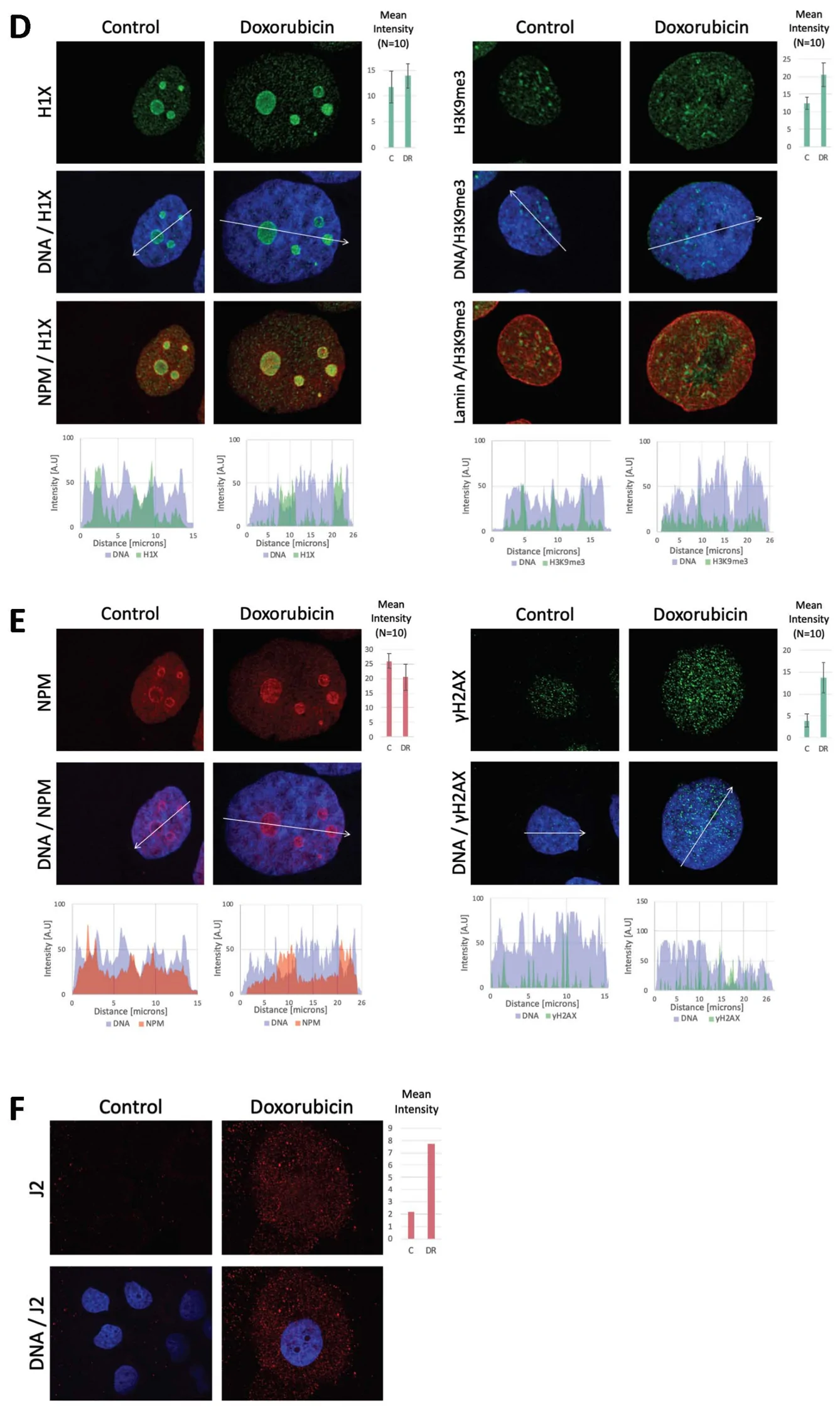
H1 variant distribution in the nuclei of T47D cells treated with doxorubicin. Confocal immunofluorescence analysis of H1.0, H1.2, H1.3, H1.4, H1.5, H1X, Lamin A, Nucleophosmin (NPM), J2 (binding to cytosolic dsRNA), γH2AX and H3K9me3 (green or red), and DNA staining (blue) in T47D cells without and with doxorubicin 0.05 µM treatment for 5 days. Bottom panel shows the plot of intensity profile of the molecule and the DNA staining along the depicted white arrow. Intensity plots next to images show the mean intensity of the signal within the nucleus for *N* = 10 in control (**C**) and treated cells (DR), with error bars depicting the standard deviation of the measurements. The following markers were used: (**A**) Left, Lamin A (red) and DNA. Right, H1.0 (red) and DNA. (**B**) Left, H1.2 (green), Lamin A (red) and DNA. Right, H1.3 (green), Lamin A (red) and DNA. (**C**) Left, H1.4 (green), H1.0 (red) and DNA. Right, H1.5 (green), Lamin A (red) and DNA. (**D**) Left, H1X (green), NPM (red) and DNA. Right, H3K9me3 (green), Lamin A (red) and DNA. (**E**) Left, NPM (red) and DNA. Right, γH2AX (green) and DNA. (**F**) J2 for dsRNA (red) and DNA.

Immunofluorescence imaging, depicted in Figure 2, shows that the signal of the trimethylation of lysine 9 in histone 3 (H3K9me3) associated with heterochromatin is spread through the nucleus of control cells. In the senescent cells (DoxoR-treated), a stronger signal was recorded in the nuclear periphery along the nuclear lamina, and also, throughout the nucleus, the signal was more intense than in the control cells, compatible with the formation of senescence-associated heterochromatin foci (SAHF). The nuclear area was also expanded, and Lamin A showed some misplacement (Figure 2). Additionally, the γH2AX signal, indicating DNA damage, was universally distributed throughout the nucleus in both control and senescent cells, but it appears to have a stronger intensity in the latter. Overall, several senescence markers showed an increase upon drug treatment.

### 3.2. Upregulation of H1.0 in Senescence Induced Cells

Next, we analyzed whether the expression, accumulation or distribution of histone H1 variants was affected by senescence induction. The expression of the genes for the different H1 variants was assessed by RT-qPCR with variant-specific primer pairs. *GAPDH* expression was used as a control (housekeeping gene). The results of this study not only show a significant upregulation of H1.0 in RT-qPCR on a transcriptional level in DoxoR-treated T47D cells (Figure 1B and Figure S2), but also on a protein accumulation level depicted in the Coomassie-stained gels, as well as in the Western blot (Figure 1C,D and Figure S3). This is additionally supported by a higher signal intensity of H1.0 in the immunofluorescence imaging (Figure 2). Treatment with etoposide and alisertib did not result in increased H1.0 transcription; however, a moderate upregulation at the protein level was observed in the SDS–PAGE and Western blot analysis (Figure 1).

### 3.3. Loss of Peripheral H1.2 Enrichment in Senescence-Induced Cells

The immunofluorescence imaging results of this study show a peripheral enrichment of H1.2 in the control cells as reported before [23], whereas the distribution greatly varies in the senescence-induced cell. In the senescent cell, H1.2 accumulates more in the center of the nucleus, seemingly surrounding the nucleoli with an enrichment in denser chromatin regions based on the matching pattern of DNA and H1.2 staining (Figure 2). The significant decrease of H1.2 in the RT-qPCR results, as well as the Coomassie staining, suggest a lower H1.2 content in all treated conditions (Figure 1B,C and Figure S2). On the other hand, the Western blot results show some upregulation in alisertib and etoposide-treated cells (Figure 1D and Figure S3). Although it remains uncertain whether H1.2 protein is decreased in senescence-induced cells, the data clearly shows a redistribution of the protein within the nucleus.

### 3.4. Deregulation of H1.3, H1.4, H1.5, and H1X in Senescence-Induced Cells

This study shows a significant decrease of H1.3, H1.4, H1.5 and H1X on the RNA level upon senescence induction with the three drugs (Figure 1B and Figure S2). This tendency is also observed on the protein level by a lower intensity of the band combining H1.3, H1.4 and H1.5 in the Coomassie staining (Figure 1C). According to previous experience from the lab, due to their similar molecular weight, the H1 variants H1.3, H1.4 and H1.5 appear in one band, while H1.2 and H1.0 have a distinguishable lower weight. Due to its low contribution to the overall H1 content and properties that change its behavior in the SDS–PAGE run, H1X is not represented in that band. Western blotting also suggests slightly decreased levels of H1.3, H1.4 and H1X, at least upon senescence induction (Figure 1D and Figure S3). H1.1 is not expressed in T47D cells and remains undetectable after drug treatment.

Within the control cells, H1.3 and H1.5 show abundance throughout the nucleus with an enrichment towards the peripheral regions close to the lamina (Figure 2). In senescence-induced cells, H1.3 especially seems to be downregulated and furthermore losing its peripheral enrichment according to Western blot and immunofluorescence. For H1.5 on the other hand, the Western blot does not indicate a downregulation, and immunofluorescence imaging only shows a slight decrease in signal intensity throughout the enlarged nucleus in DoxoR-treated cells, while it still shows a higher abundancy towards the nuclear lamina.

In the present study, a decrease of H1.4 in senescent cells is observed in the Western blot, as well as in the immunofluorescence imaging (Figure 1D and Figure 2). H1.4 is localized throughout the nucleus, showing the same distribution pattern within control and treated cells, although the signal intensity was lower in the latter.

H1X shows a slight decrease on a protein level in the Western blot but a higher signal intensity in the immunofluorescence imaging in senescent cells (Figure 1D and Figure 2). It is mostly located in the nucleoli in the control (as reported before; Ref. [23]), as well as in the treated cells. Furthermore, within the nucleoli of the control cell, H1X seems to be enriched at the nucleolar rim, aligning with the signal of nucleophosmin (NPM).

Overall, the RT-qPCR and immunofluorescence imaging results of the present study provide evidence for a decrease of H1.3, H1.4 and H1.5 in senescent cells, whereas the results for H1X are not conclusive. The degree of downregulation varies among the H1 variants.

### 3.5. Increased Transcription of TEs and ISGs and Accumulation of Cytosolic dsRNA in Senescence-Induced Cells

It has been reported that in senescence, transposable element (TE) expression and the activation of the interferon (IFN) response occur [11,13,14,30]. In addition, this also occurs upon depletion of particular H1 variants in T47D cells, i.e., the simultaneous knock-down of H1.2 and H1.4, but not other H1 variant combinations [24]. Chromatin decompaction allows for the derepression of TEs, the accumulation of cytosolic RNAs, and the activation of the IFN response, depicted by the upregulation of many IFN-stimulated genes (ISGs). In order to further correlate senescence induction, H1 variant changes, and TE and ISG upregulation, we investigated this in senescence-induced T47D cells.

To investigate TE transcriptional regulation, we targeted 16 distinct sequences representing major TE families. As depicted in Figure 1E and Figure S2, DoxoR treatment triggered widespread TE derepression, with the most pronounced increases occurring in LINE, SINE and satellites. A highly concordant pattern was observed upon etoposide treatment, which shows a certain upregulation of TEs across LINE, SINE, satellite, and LTR families. By comparison, alisertib treatment elicited a more subdued and variable transcriptional response: most tested TEs remained unchanged, one subset showed mild repression, and only *MLT1J2* exhibited significant upregulation (Figure 1E).

Candidate ISGs were selected for RT-qPCR analysis based on prior dataset observations, the published literature, and validated primer sets. As depicted in Figure 1F and Figure S2, the induction of senescence elicited a significant upregulation of *OASL* transcription across all treated groups relative to the untreated controls. A similar global elevation was observed for *IFIT3*, whereas a significant induction of *IFI27* was confined to DoxoR treatment. Conversely, *IFIT2* exhibited a slight, non-significant elevation exclusively in DoxoR-treated cells, and *IFI6* levels remained comparable across all experimental conditions. Taken together, this data indicates that ISG expression is biased toward transcriptional upregulation upon senescence induction.

Moreover, immunofluorescence imaging of senescent cells showed a marked accumulation of cytosolic double-stranded RNA (dsRNA) detected by the J2 monoclonal antibody (Figure 2F). This increase in cytoplasmic nucleic acids supports a model of transposable element (TE) reactivation driving subsequent interferon (IFN) pathway activation, echoing the mechanism triggered by simultaneous depletion of multiple H1 variants [24].

### 3.6. Effect of H1 Depletion on Senescence Induction

**Figure 3:**
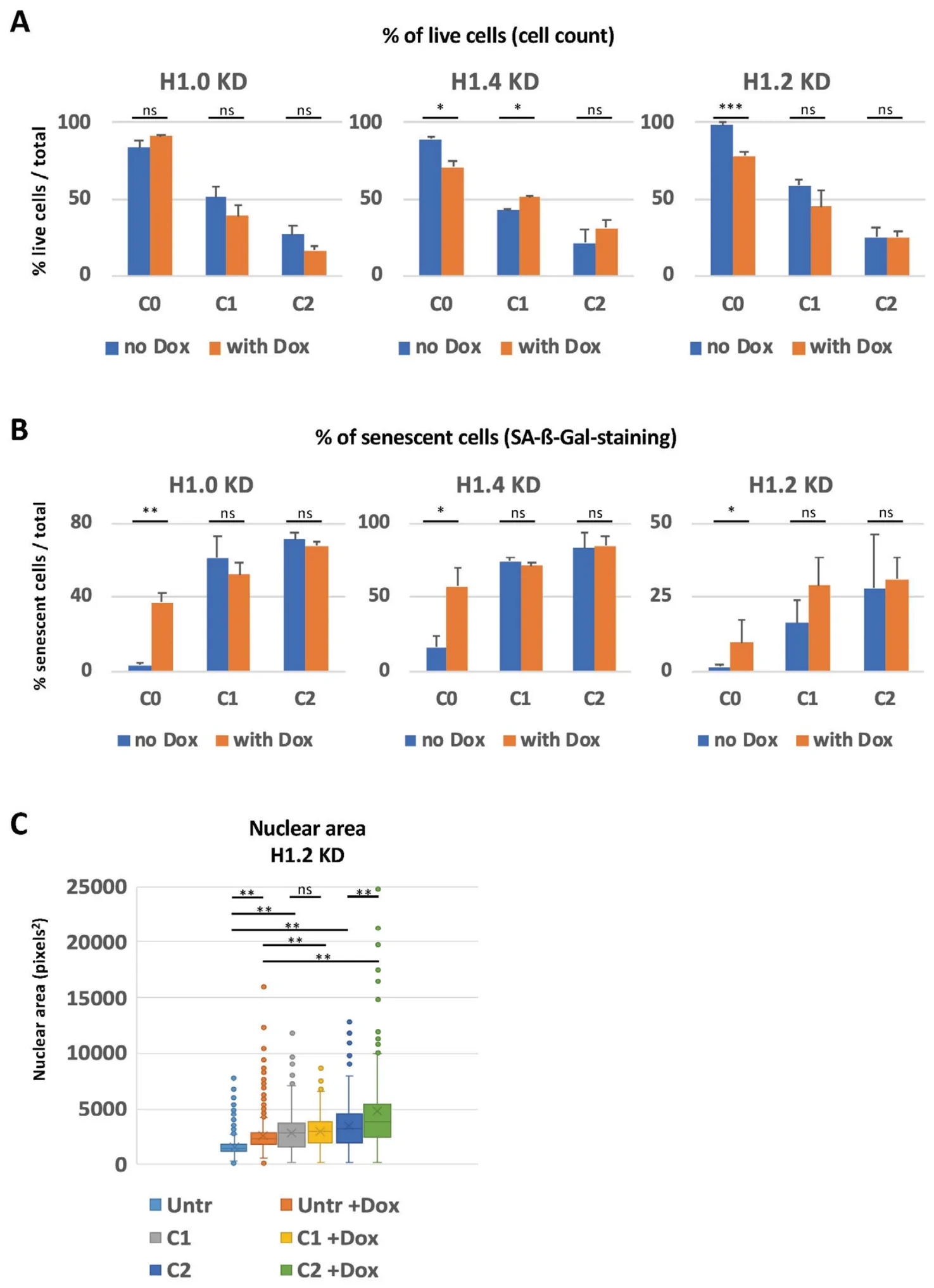
Effect of H1 variant depletion on drug-induced senescence. T47D cells harboring a doxycycline-inducible lentivirus for the knock-down (KD) of H1.0, H1.2 or H1.4 were treated with doxycycline (±Dox) for 6 days to induce H1 KD and with two different concentrations of doxorubicin (DoxoR C1 = 0.01 and C2 = 0.05 µM) for 5 days or left untreated. (**A**) After staining with trypan blue, dead and live cells were counted and expressed as percentage of live cells. (**B**) After staining for ß-galactosidase, the percentage of senescent cells was calculated. (**A**,**B**) A representative experiment analyzed in triplicate is shown. Statistical differences of ±Dox (H1 KD) are supported by *t*-test. (***) *p*-value ≤ 0.005; (**) *p*-value ≤ 0.01; (*) *p*-value ≤ 0.05; (ns/non-significant) *p*-value > 0.05. Another representative experiment for (**A**) is shown as Supplementary Figure S4. (**C**) Nuclear area of H1.2 KD T47D cells treated with Dox and DoxoR as in (**A**,**B**) and stained with Hoechst. Microscope images (*N* > 190 per condition) were analyzed with ImageJ, and nuclear areas (square pixels) are represented as boxplot. Statistical differences ±Dox (H1 KD) or ±DoxoR are supported by *t*-test. (**) *p*-value ≤ 0.01; (ns/non-significant) *p*-value > 0.05.

In order to investigate whether the depletion of H1 variants alters the induction of senescence by topoisomerase inhibitors, we treated doxycycline (Dox)-inducible H1 KD T47D cells (see Section 2) with two different concentrations of DoxoR; cell growth was followed by cell counting (live and dead cells), and cell senescence was followed by ß-galactosidase staining. H1 KD by itself reduced the number of cells in the case of H1.2 and H1.4, but not H1.0 KD (Figure 3A), as previously reported [24,27]. On the other hand, DoxoR also reduced the number of cells drastically and dose-dependently in WT cells (Dox-untreated). Upon H1.4 KD (Dox-treated), the effect of DoxoR was less drastic than in WT cells, i.e., the percentage of live cells decreased less pronouncedly. For H1.0 KD, it was the opposite (Figure 3A and Figure S4).

Then, we investigated the induction of senescence by topoisomerase inhibitors and H1 depletion by measuring SA-ß-Gal staining. In the absence of DoxoR, all H1 KDs promoted some cells to become senescent, without reaching the effect caused by drug treatment (Figure 3B). DoxoR treatment induced the accumulation of senescent cells to similar levels in WT and H1 KD conditions (±Dox). In other words, H1 variant KD did not enhance DoxoR-induced senescence. Because nuclear area increase may be indicative of cellular senescence upon drug treatment, we measured the nuclear area of H1.2 KD cells treated with ±Dox to induce KD and ±DoxoR (Figure 3C). As with the SA-ß-Gal staining, we observed significant increases in nuclear area upon H1.2 KD and DoxoR treatment. At high DoxoR concentration, H1.2 KD further increased nuclear areas, suggestive of additive effects on senescence induction.

**Figure 4:**
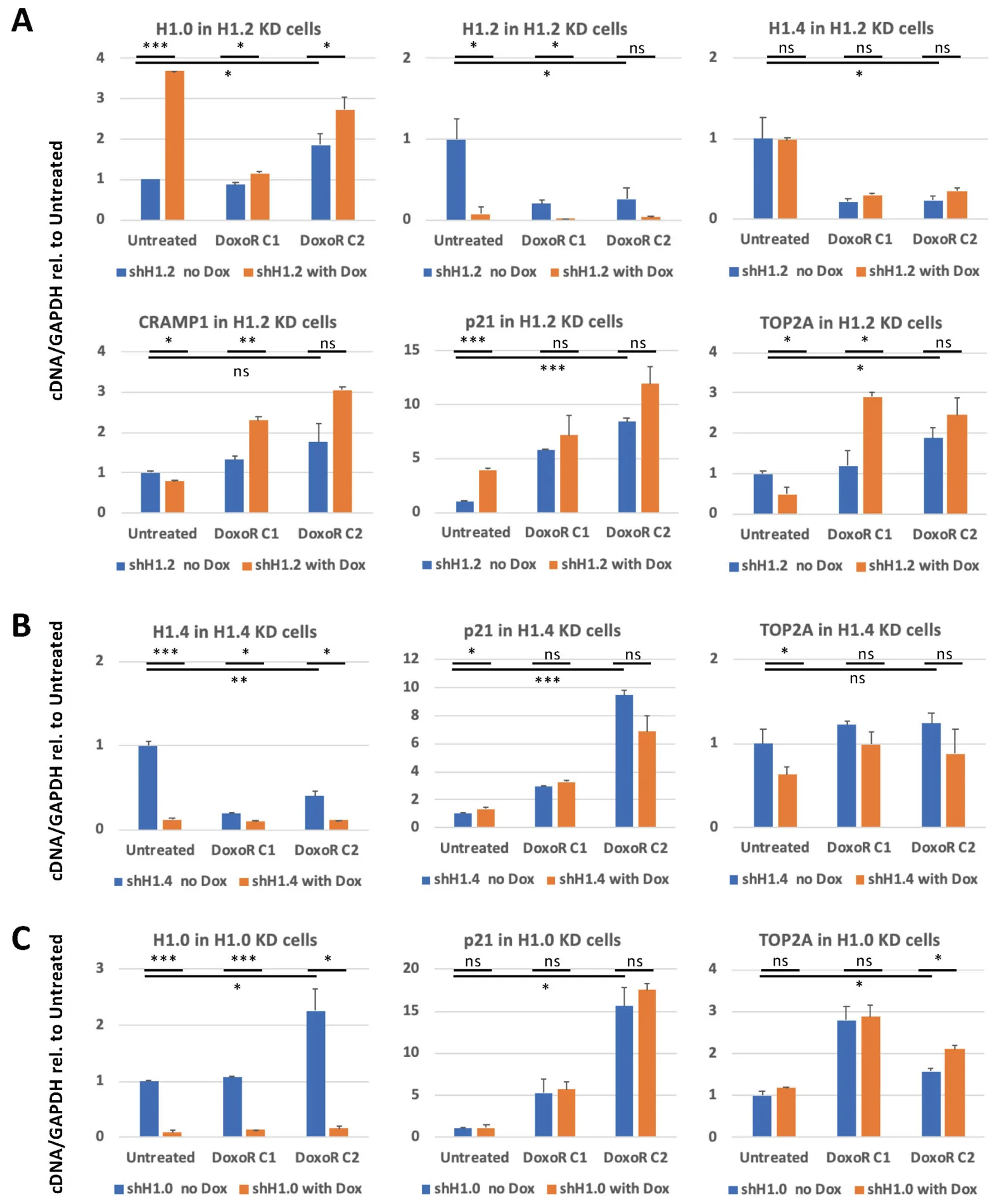
Effect of H1 variant depletion and doxorubicin treatment on gene expression. T47D cells harboring the inducible system for H1.2 (**A**), H1.4 (**B**) or H1.0 (**C**) KDs were treated with ±Dox and ±DoxoR C1 or C2, as seen in Figure 3, and, after RNA extraction, gene expression was measured by RT-qPCR for the indicated genes. GAPDH gene was used for normalization, and data are expressed relative to the untreated sample. Representative experiments analyzed in triplicate are shown. Additional representative experiments are shown in Supplementary Figure S5. Statistical differences ±Dox (H1 KD) or ±DoxoR are supported by *t*-test. (***) *p*-value ≤ 0.005; (**) *p*-value ≤ 0.01; (*) *p*-value ≤ 0.05; (ns/non-significant) *p*-value > 0.05.

In order to investigate whether the depletion of H1 variants alters changes in gene expression caused by topoisomerase inhibitors, we treated inducible H1 KD cells with two different concentrations of DoxoR and measured the gene expression by RT-qPCR. As mentioned above, in WT cells (-Dox), DoxoR caused the downregulation of replication-dependent H1 variant genes, such as H1.2 and H1.4, and the upregulation of H1.0, *p*21^CIP1/WAF1^, and *Top2A* to some extent, as well as *CRAMP1*, a positive regulator of H1 gene expression [31,32]. In H1 KD conditions, the transcriptional effects of DoxoR were overall maintained (Figure 4A–C and Figure S5).

**Figure 5:**
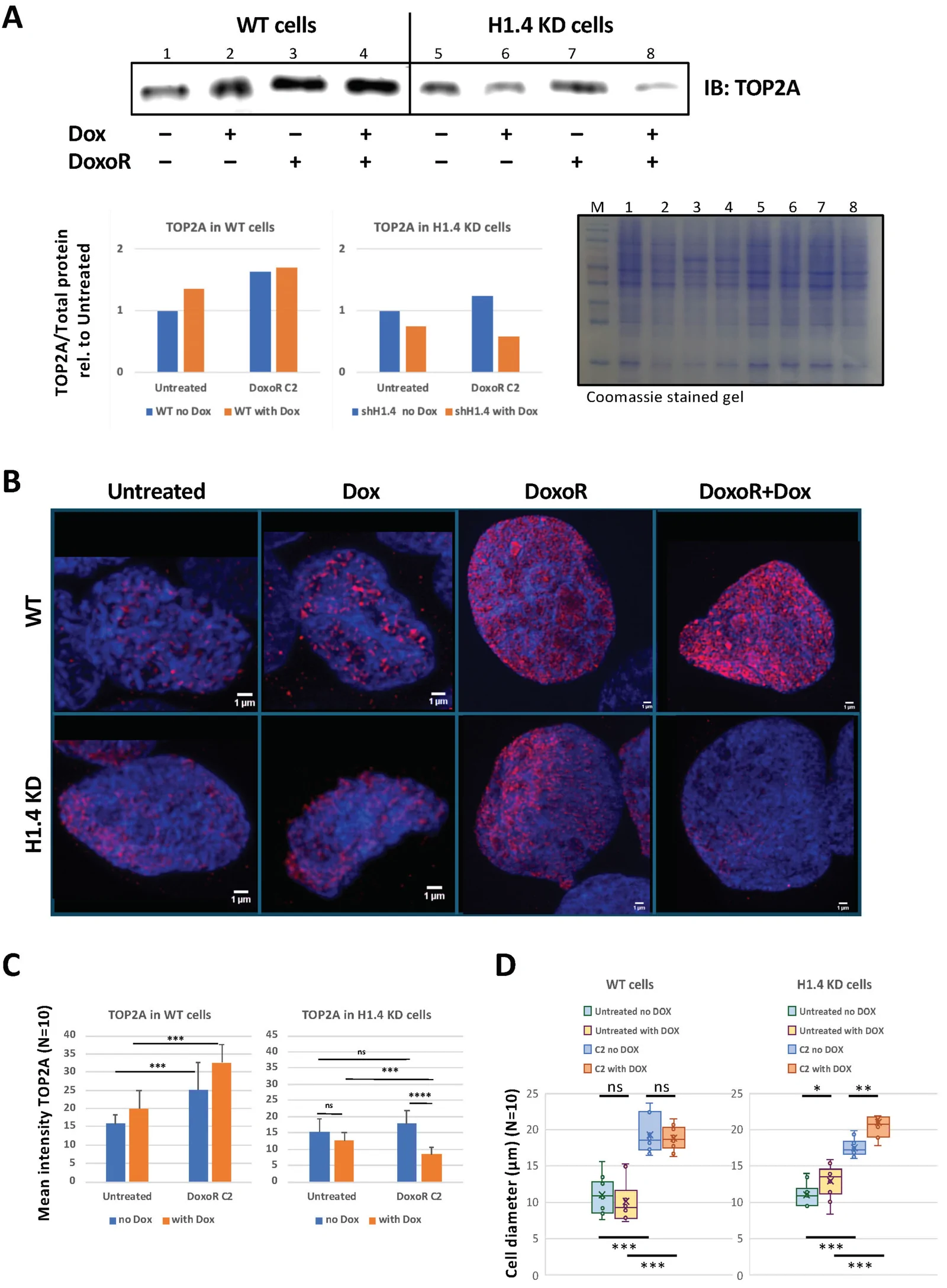
Response of TOP2A to doxorubicin and H1.4 knock-down. (**A**–**C**) WT and H1.4 KD cells were treated with ±Dox and ±DoxoR C2 and submitted to immunoblotting (**A**) or immunofluorescence (**B**) with antibody-recognizing TOP2A. (**A**) Immunoblotting bands were quantified, normalized to total protein (Coomassie staining bands, right panel) and represented relative to the untreated sample. (**B**,**C**) Immunoblotted cells were stained with DAPI (blue). TOP2A intensity was quantified and represented (*N* = 10 cells/condition). Statistical differences are supported by *t*-test. (****) *p*-value ≤ 0.0001; (***) *p*-value ≤ 0.001; (**) *p*-value ≤ 0.01; (*) *p*-value ≤ 0.05; (ns/non-significant) *p*-value > 0.05. One µm-scale bars are shown for each representative image. (**D**) The cell diameter of immunoblotted cells is represented (in microns) for each condition (*N* = 10 cells/condition) in the boxplots. Statistical differences of ±Dox (H1 KD) or ±DoxoR are supported by *t*-test. (***) *p*-value ≤ 0.001; (**) *p*-value ≤ 0.01; (*) *p*-value ≤ 0.05; (ns/non-significant) *p*-value > 0.05. Plots showing the intensity profile of TOP2A and DNA staining along the depicted line used to measure the diameter are shown as Supplementary Figure S6 for two representative cells per condition.

H1.2 KD by itself promoted the upregulation of *p*21^CIP1/WAF1^, but this did not occur upon H1.4 or H1.0 KD, as well as the upregulation of H1.0 as reported before [24,27]. Upon H1.4 KD, *Top2A* was downregulated—observed by RT-qPCR, immunoblotting, and immunofluorescence—both in the absence or presence of DoxoR (Figure 4B,  Figure S5 and Figure 5A–C). As mentioned above, DoxoR treatment induced *Top2A* expression and protein accumulation, both in WT and uninduced (-Dox) H1 KD cells. Instead, the addition of DoxoR on H1.4 KD conditions decreased TOP2A protein accumulation (Figure 5A–C). TOP2A and DNA intensity profiles across nuclei are shown in Supplementary Figure S6 to further depict the effect of H1.4 KD on the induction of TOP2A in response to DoxoR. Utilizing these immunofluorescence experiments, the nuclei diameters were measured in all conditions (Figure 5D). As indicated above, DoxoR-induced senescence promoted a nuclei size increase in all cell lines tested. Interestingly, H1.4 KD (+Dox) also increased nuclei size to some extent (Figure 5D), in agreement with the increase of senescence described above for this H1 KD (Figure 3B). As for H1.2 KD, H1.4 KD and DoxoR effects on nuclear size were additive.

**Figure 6:**
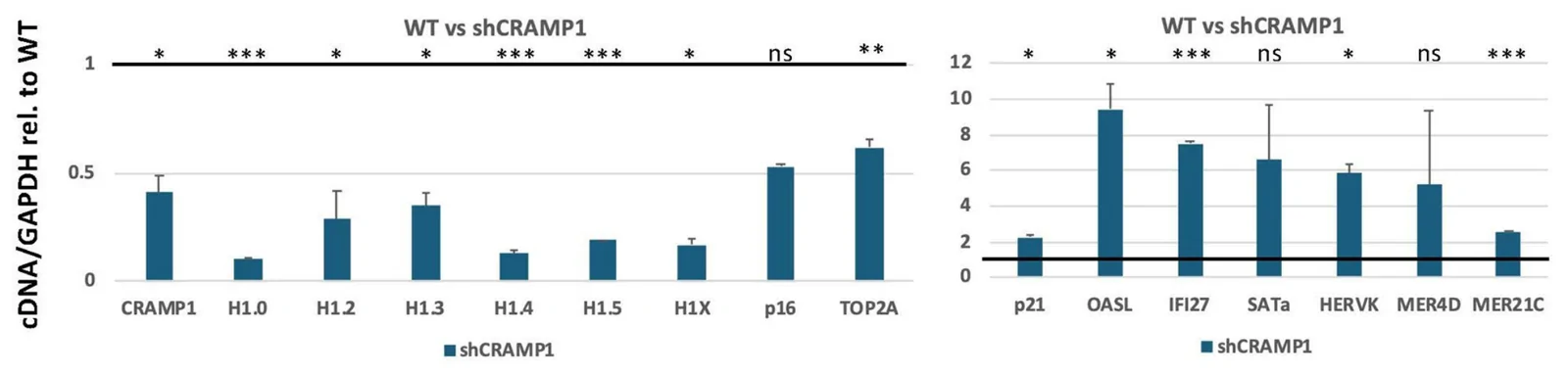
Effect of CRAMP1 depletion on the expression of H1 variants, ISGs and TEs. Wild-type (WT) and CRAMP1 KD T47D cells were submitted to RNA extraction, and gene expression was measured by RT-qPCR for the indicated genes (CRAMP1, H1 variants, p16^INK4a^, p21^CIP1/WAF1^, Top2A, OASL and IFI27) and transposable elements. GAPDH gene was used for normalization and data is expressed as fold-change relative to WT cells. Left panel shows downregulated genes in shCRAMP1 compared to WT (FC < 1, black line); right panel shows upregulated genes or TEs (FC > 1, black line). A representative experiment analyzed in triplicate is shown. Statistical differences ±shCRAMP1 are supported by *t*-test. (***) *p*-value ≤ 0.005; (**) *p*-value ≤ 0.01; (*) *p*-value ≤ 0.05; (ns/non-significant) *p*-value > 0.05.

Another way to investigate the effects of general H1 depletion is to knock down CRAMP1, a positive regulator of H1 gene expression [31,32]. T47D cells were infected with lentiviruses for the constitutive expression of a CRAMP1 shRNA and selected with puromycin for 7 days before RNA extraction. RT-qPCR confirmed the depletion of *CRAMP1* and the expected downregulation of all histone H1 variant genes (Figure 6). In parallel, the *p*16^INK4a^ gene was downregulated and *p*21^CIP1/WAF1^ was upregulated, which could indicate cell senescence but also other cell cycle or proliferation effects. *Top2A* was also downregulated, as shown in H1.2 and H1.4 KD conditions. The expression of two interferon-stimulated genes (ISGs) was also tested. Upon CRAMP1 KD, *OASL* and *IFI27* genes were highly upregulated (Figure 6). The induction of ISGs has been described before upon KD of multiple H1 variants [24], but also in senescent cells [11,13,14,30], and could be due to the deregulation of transposable elements (TEs) or satellites. In accordance, upon CRAMP1 KD, the upregulation of *SATa*, *HERVK*, *MER4D* and *MERC21C* was observed, which were either statistically significant or not significant (Figure 6). Further testing of senescence induction upon stable depletion of CRAMP1 will help in the future to confirm the importance of histone H1 in preventing senescence.

Overall, we have shown that drugs inducing senescence alter the stoichiometry of H1 variants, which may erode heterochromatin at repetitive loci, driving TE expression, dsRNA accumulation and an ISG-mediated cascade. Because these effects are also seen in T47D cells by the downregulation (via CRAMP1 KD) or knock down of certain H1 variants (herein and [24]), we propose that H1 variants play a significant role in the changes occurring in heterochromatin along senescence induction and that maintaining optimal H1 variant stoichiometry may be required to prevent spontaneous senescence. This model will be further tested in the future using additional tumoral and non-tumoral cell types.

## 4. Discussion

In the present study, we propose that therapy-induced senescence (TIS) in T47D breast cancer cells—triggered by genotoxic agents (doxorubicin, etoposide) or mitotic inhibition (alisertib)—may be driven by a coordinated interplay between chromatin remodeling, linker histone redistribution, transposable element reactivation, and innate immune pathway induction.

The treatment of T47D cells with optimized low-dose drug regimens successfully induced classical markers of cellular senescence, including positive SA-ß-Gal activity, increased cell and nuclear area, elevated *p*21^CIP1/WAF1^ expression, and the intensification of gamma-H2AX foci reflecting persistent double-strand breaks. Furthermore, the redistribution of H3K9me3 toward the nuclear periphery along with Lamin A misplacement aligns with the spatial reorganization of chromatin into senescence-associated heterochromatin foci (SAHF), a hallmark mechanism for permanently silencing pro-proliferative genes (such as E2F targets) and reinforcing cell cycle arrest [2,3,4,5].

It has been described elsewhere that in oncogene-induced senescent fibroblasts, SAHF formation is accompanied by a loss of histone H1 and increased chromatin-bound high-mobility group A2 (HMGA2), a competitor protein for linker DNA [25]. Because no distinction among H1 variants was reported, we decided to investigate the dynamics of H1 variant expression and localization upon senescence induction. Our findings reveal a shift in the composition of the linker histone pool during TIS. We observed a robust upregulation of the replication-independent variant H1.0 at both the mRNA and protein levels. As H1.0 is associated with terminally differentiated, non-dividing states, its accumulation likely drives the hyper-compaction of heterochromatic domains and reinforces long-term proliferation arrest. Conversely, the transcript levels for replication-dependent H1 variants (H1.2–H1.5) declined, reflecting the shutdown of canonical, replication-dependent histone gene transcription upon cell cycle exit. However, retained protein pools of variants such as H1.2 and H1.4—due to their long half-lives in non-dividing cells—may remain critical for stabilizing SAHF architecture and maintaining structural integrity at the nuclear lamina. The replication-independent variant H1X maintained steady expression and nuclear localized dynamics, consistent with its specialized roles at active gene promoters [23,28].

Despite global chromatin compaction, drug treatment—particularly with genotoxic topoisomerase inhibitors—triggered prominent derepression of multiple transposable element (TE) classes, including LINEs (e.g., *L1PA3*, *L1HS*), SINEs (e.g., *Alu* elements), SVAs, and LTRs (e.g., *EnvE*, *MLT1J2*). This localized loss of heterochromatic silencing mirrors the phenotypes observed upon multi-variant H1 knock down [24], suggesting that chemotherapeutic stress destabilizes linker histone-mediated silencing at repetitive genomic regions. Variants such as H1.2 and H1.4, in coordination, are important for maintaining heterochromatic TE repression, and its depletion or downregulation—as in senescent cells—is not sufficiently compensated by H1.0 accumulation. H1.2, H1.3 and H1.5 are enriched at the nuclear periphery and lamina-associated domains (LADs) in T47D cells, co-localizing with dense H3K9me2/3 domains [23]. Instead, H1.4 is localized within the nuclei without preference for the periphery but is known to be post-translationally modified (such as H1.4K26 methylation) to act as a docking site for HP1 and chromatin modifiers that may consolidate heterochromatic foci [33]. Additionally, linker histones have been found to be enriched at H3K27me2-marked genomic loci and to participate in Polycomb repression [34], but no variant specificity has been described so far there. Overall, the similarity between the TE reactivation profile in drug-treated T47D cells and H1-depleted cells suggests that drug-induced chromatin remodeling may impair the ability of H1 variants to maintain repetitive element silencing, even as the total H1.0 accumulates.

Critically, the bidirectional transcription of these derepressed retrotransposons may result in the accumulation of cytosolic double-stranded RNA, as confirmed by high J2 immunofluorescence signals. Cytoplasmic dsRNA is known to serve as an endogenous ligand for pattern-recognition receptors (PRRs), initiating a state of viral mimicry that links epigenetic instability directly to innate immune signaling. As a downstream consequence of cytosolic dsRNA sensing, senescent cells may activate an interferon-stimulated gene (ISG) response. *OASL* and *IFIT3* were consistently upregulated across all senescence-inducing treatments. *IFI27* exhibited significant induction under specific drug conditions (doxorubicin and alisertib), whereas *IFIT2* and *IFI6* remained largely unaffected. This specificity demonstrates that senescence may trigger a targeted, rather than generalized, ISG program to modulate cell-intrinsic immune surveillance. It has been described before that during cellular senescence, L1 (also known as LINE-1) retro-transposable elements become transcriptionally derepressed and activate a type-I interferon (IFN-I) response [30]. The IFN-I response was triggered by cytoplasmic L1 cDNA and was antagonized by inhibitors of the L1 reverse transcriptase. As multiple TE classes have been found to be activated in our study, it cannot be discarded that the ISG response may be triggered by several recognition mechanisms elicited by dsRNA or cDNAs, which would deserve further investigation.

To determine whether alterations in linker histone dynamics are causal drivers or downstream consequences of senescence, we investigated the functional impact of single-variant knock downs (H1.0, H1.2, H1.4) and global H1 depletion via CRAMP1 knock down in T47D breast cancer cells, both at baseline and in combination with doxorubicin. The knock down of individual H1 variants yielded distinct cellular phenotypes, highlighting functional non-redundancy among somatic linker histones.

In agreement with previous reports [24,27], the depletion of H1.2 or H1.4 in untreated cells markedly reduced cell proliferation and live cell counts. We observed that single-variant depletion can autonomously initiate a senescent phenotype in the absence of drug treatment. The baseline knock down of H1.0, H1.2 or H1.4 promoted SA-ß-Gal positivity. When combined with DoxoR treatment, no H1 variant depletion further elevated the maximum percentage of senescent cells (as measured by SA-ß-Gal staining) relative to DoxoR-treated WT controls. This saturation effect suggests that genotoxic stress and linker histone depletion act through convergent downstream pathways (such as *p*21^CIP1/WAF1^ upregulation and chromatin restructuring) to enforce cell cycle exit. Instead, by measuring nuclear size, H1.2 and H1.4 KD showed an additive effect with DoxoR, so further studies need to be developed to clarify whether topoisomerase inhibition and H1 variant KD act through convergent mechanisms leading to senescence.

Analyzing gene expression shifts across H1 KD models revealed intricate regulatory feedback loops. H1.2 KD selectively upregulated H1.0 and *p*21^CIP1/WAF1^ mRNA, mimicking aspects of the DoxoR-induced transcriptional signature. This indicates that chromatin relaxation resulting from H1.2 loss is sufficient to derepress key stress-response genes. While DoxoR induces *Top2A* transcription across conditions, H1.4 KD specifically caused a striking reduction in Top2A protein levels (observed via RT-qPCR, immunoblotting, and immunofluorescence). Upon DoxoR addition in H1.4 KD cells, *Top2A* mRNA was transcribed, but Top2A protein accumulation was blunted. Given that topoisomerase II alpha (Top2A) is the direct molecular target of DoxoR, this H1.4-dependent post-transcriptional or protein stability defect of Top2A may generate a complicated interplay when DoxoR and H1.4 KD are combined.

To evaluate the impact of global H1 downregulation without single-variant compensation, we knocked down CRAMP1, a positive regulator of linker histone gene expression [31,32]. Constitutive shRNA-mediated depletion of CRAMP1 resulted in the simultaneous downregulation of all somatic H1 variants, at least at the mRNA level (Figure 6). Pan-H1 suppression by CRAMP1 KD downregulated *p*16^INK4a^ while significantly upregulating *p*21^CIP1/WAF1^, aligning with a p21-driven senescence entry. CRAMP1 depletion led to Top2A downregulation and altered the expression of ISGs. This confirms that the broad disruption of H1 dosage triggers both cell cycle arrest and an innate immune response, reinforcing the concept that chromatin unwrapping caused by H1 loss may serve as an endogenous trigger for ISG activation and, potentially, viral mimicry.

## 5. Conclusions

Taken together, our data suggests a unified model wherein drug-induced senescence induces a major remodeling of linker histone stoichiometry dominated by H1.0 accumulation, but also the downregulation and mislocalization of the replication-dependent variants. This epigenetic transition may lead to localized heterochromatin erosion at repetitive loci, triggering retrotransposon expression, cytosolic dsRNA accumulation, and ISG activation. Targeting this axis could provide new therapeutic strategies to enhance the immune recognition of senescent tumor cells. This non-canonical immune response reinforces cell cycle arrest and primes senescent cancer cells for immune surveillance, representing a major mechanism through which chemotherapeutics alter tumor-cell intrinsic signaling beyond simple cell-cycle inhibition.

Beyond documenting correlation, our functional depletion experiments demonstrate that linker histones actively modulate the threshold for senescence induction and genotoxic response. Single-variant knock downs revealed distinct roles. To validate these findings under global H1 restriction, the shRNA depletion of CRAMP1 simultaneously suppressed all H1 variants, downregulated *Top2A*, and activated *p*21^CIP1/WAF1^ along with downstream ISGs. Together, these depletion models suggest that maintaining optimal H1 variant stoichiometry may be required to prevent spontaneous senescence, whereas global H1 depletion via CRAMP1 loss may cause p21-mediated cell cycle arrest and innate immune signaling.

## Data Availability

Data is contained within the article or Supplementary Materials.

## Supplementary Materials

The following supporting information can be downloaded at https://www.mdpi.com/article/10.3390/genes17101274/s1, Figure S1: β-galactosidase staining of T47D control and senescence induced cells. (A) Cells treated with doxorubicin (DR) 0.05 μM, 0.1 μM and 0.5 μM. (B) Cells treated with alisertib (AL) 0.5 μM, 1 μM and 3 μM. (C) Cells treated with etoposide (ET) 0.1 μM, 0.5 μM and 3 μM. Staining and imaging after two and five days. Red asterisks mark the conditions further used along this study. Related to Figure 1; Figure S2: Effects of doxorubicin on the expression of H1 variant genes, senescence markers, ISGs and TEs. T47D-derived cells harboring shRNA for H1.2 (A) or H1.4 (B) KD (without doxycycline) were treated for five days with doxorubicin (DoxoR) 0.05 μM or left untreated (control), and gene expression was measured by RT-qPCR in triplicate with oligonucleotides specific for p21^CIP1/WAF1^ and p16^INK4a^, six H1 variant genes, transposable elements of five classes, or five ISGs, as in Figure 1. GAPDH gene was used for normalization and data expressed as fold-change relative to untreated control (dashed line marks foldchange = 1). Statistical differences ±DoxoR are supported by *t*-test. (***) *p*-value ≤ 0.005; (**) *p* value ≤ 0.01; (*) *p*-value ≤ 0.05; (ns/non-significant) *p*-value > 0.05. Related to Figure 1; Figure S3: Immunoblotting of H1 variants and H3K9me3 upon senescence induction. (A) Immunoblotting of H1 variants H1.1 to H1.5, H1X and the PTM H3K9me3 on seven different membranes, with histone 3 (H3) blotting as loading control and Coomassie staining of the SDS-PAGE gel after protein transfer to the membrane. T47D cells were untreated (C) or treated for 5 days with doxorubicin 0.05 μM (DR), alisertib 0.5 μM (AL) and etoposide 0.5 μM (ET) and total histones extracted. (B) Immunoblotting of H1.0 on six of the seven membranes used in (A) due to the compatibility of secondary antibodies raised against rabbit (H1 variants) or mouse (H1.0) primary antibodies. Molecular weight marker (M) is shown. Related to Figure 1D; Figure S4: Effect of H1 variant depletion and drug-induced senescence on cell viability. T47D cells harboring a doxycycline-inducible lentivirus for the knock-down (KD) of H1.0, H1.2 or H1.4 were treated with doxycycline (±Dox) for 6 days to induce H1 KD and with two different concentrations of doxorubicin (DoxoR C1 = 0.01 and C2 = 0.05 μM) for 5 days or left untreated. Cells were analyzed by FACS and gated as live cells in the FSC and SSC scatter plot to distinguish them from debris or enlarged cells. Results are expressed as % of live cells. Related to Figure 3A; Figure S5: Effect of H1 variant depletion and doxorubicin treatment on gene expression. T47D cells harboring the inducible system for H1.2 (A), H1.4 (B) or H1.0 (C) KDs were treated ± Dox and ±DoxoR C1 or C2 as in Figure 3, after RNA extraction, gene expression was measured by RT-qPCR for the indicated genes. GAPDH gene was used for normalization and data expressed relative to the untreated sample. Representative experiments analyzed in triplicate are shown. Statistical differences ±Dox (H1 KD) or ±DoxoR are supported by *t*-test. (***) *p*-value ≤ 0.005; (**) *p*-value ≤ 0.01; (*) *p*-value ≤ 0.05; (ns/non-significant) *p*-value > 0.05. Related to Figure 4; Figure S6: Intensity profile of TOP2A and DNA staining in WT and H1.4 KD cells treated with doxorubicin. WT and H1.4 KD cells were treated ± Dox and ±DoxoR C2 and submitted to immunofluorescence with antibody recognizing TOP2A and DAPI stained. Plots show the intensity profile of TOP2A (in red) and DNA (in blue) staining along a depicted line used to measure the cell diameter for two representative cells per condition. Related to Figure 5B–D; Table S1: Oligonucleotide sequences used for RT-qPCR; Table S2: Immunostaining antibody combinations.

## Abbreviations

The following abbreviations are used in this manuscript:

SASP	Senescence-associated secretory phenotype
SAHF	Senescence-associated heterochromatin foci
TE	Transposable element
ISG	Interferon-stimulated gene
TIS	Therapy-induced senescence
AL	Alisertib
ET	Etoposide
Dox	Doxycycline
DR or DoxoR	Doxorubicin
KD	Knock-down
SA-β-gal	Senescence-associated ß-galactosidase
IFN	Interferon
dsRNA	Double-stranded RNA
Top2A	Topoisomerase II alpha
ns	Non-significant
