# Supplementary material for: Dynamic Changes of Histone H1 Variants in Drug-Induced Senescence
Source: Genes (Basel). 2026 Oct 14;17(10):1274. doi: 10.3390/genes17101274 (PMC13704313; doi:10.3390/genes17101274)

SUPPLEMENTARY MATERIAL

Supplementary Tables

Supplementary Table S1: Oligonucleotide sequences used for RT-qPCR.

**Primers**	**Sense**	**Sequence (5' to 3')**
H1		
H1.0	F	CCTGCGGCCAAGCCCAAGCG
	R	AACTTGATCTGCGAGTCAGC
H1.2	F	GGCTGGGGGTACGCCT
	R	TTAGGTTTGGTTCCGCCC
H1.3	F	CTGCTCCACTTGCTCCTACC
	R	GCAAGCGCTTTCTTAAGC
H1.4	F	GTCGGGTTCCTTCAAACTCA
	R	CTTCTTCGCCTTCTTTGGG
H1.5	F	CATTAAGCTGGGCCTCAAGA
	R	TCACTGCCTTTTTCGCCCC
H1X	F	TTCCTTCAAGCTCAACCG
	R	TGCCTTCTTCGCTTTGTG
GAPDH		
GAPDH	F	GAGTCAACGGATTTTGGTCGT
	R	TTGATTTTGGAGGGATCTCG
Senescence Markers		
p21$^{CIP1/WAF1}$	F	AGGTGGACCTGGAGACTCTCAG
	R	TCCTCTTGGAGAAGATCAGCCG
p16^{INK4a}	F	CTCGTGCTGATGCTACTGAGGA
	R	GGTCGGCGCAGTTGGGCTCC
LINE		
L1 PA3end R (L1HS_3end F #2)	F	GGGCGAAGGACATGAACAGA
	R	CCTCTCCAGCACCTGTTGTT
L1 PA4	F	CATTTGCGGTTCACCAATATC
	R	GCTAGAGGTCCACTCCAGAC

L 1HS_5end #3 (paper #2)	F	GGCACACTGACACCTCACA
	R	AGATGGGTTTTCGGTGTGGA
SINE		
Alu Yb8 #2	F	CGAGGCGGGTGGATCATGAGGT
	R	TCTGTCGCCCAGGCCGGACT
Satellite		
SAT α	F	AAGGTCAATGGCAGAAAAGAA
	R	CAACGAAGGCCACAAGATGTC
D4Z4	F	CCGCGTCCGTCCGTGAAA
	R	TCCGTCGCCGTCCTCGTC
SST1 / NBL 2	F	AACCACTGTGACGGGAGAAA
	R	CTGGGACAGGACGAGACAC
LTR		
MER4D	F	CCCTAAAGAGGCAGGACACC
	R	TCAAGCAATCGTCAACCAGA
MER21C	F	GGAGCTTCCTGATTGGCAGA
	R	ATGTAGGGTGGCAAGCACTG
MLT1C49	F	TATTGCCGTACTGTGGGCTG
	R	TGGAACAGAGCCCTTCCTTG
HERVK	F	AGAGGAAGGAATGCCTCTTGCAG
	R	TTACAAAGCAGTATTGCTGCCCGC
LTR5_HS #1	F	AAAGGGTCTGTGCTGAGGAG
	R	AGACATTCCATTGCCCAGGG
LTR12c	F	GTCTCGCTGGCTCAGGAGTG
	R	TGAGCTGTAACACTCACCGC
EnvE	F	ACTGGCCTTTTCCTAGGTGATAC
	R	TACTATTAATGGCTGCACAAGCA
MLT1J2	F	CCTGGGTCCCTGAGTCACTA
	R	TGCCAGCTGCTGTAACAAAC
SVA		
SVA E	F	AATAGAAAGGCGGGAAGGGTG

	R	CTTCTATCCACACAGACCCGG
ISG		
IFIT2	F	ACGGTATGCTTGGAACGATTG
	R	AACCCAGAGTGTGGCTGATG
IFIT3	F	CGGAACAGCAGAGACACAGA
	R	ATGGCATTTCAGCTGTGGA
IFI6	F	CTGTGCCCATCTATCAGCAG
	R	GGGCTCCGTCACTAGACCTT
IFI27	F	TGCTCTCACCTCATCAGCAGT
	R	CACAACTCCTCCAATCACAACT
OASL	F	GGGACAGAGATGGCACTGAT
	R	AAATGCTCCTGCCTCAGAAA
other		
TOP2A	F	GTGGCAAGGATTCTGCTAGTCC
	R	ACCATTCAGGCTCAACACGCTG
CRAMP1	F	CGGATTTCTTCCTGCCAAAGCC
	R	GGACTGGTTTTCCGATGGTCTAG

Supplementary Table S2: Immunostaining antibody combinations.

No.	Target of Primary Antibody	No.	Target of Primary Antibody
1	H1.2, rabbit	5	H1X, rabbit
	Lamin A, mouse		NPM1, mouse
2	H1.3, rabbit	6	H3K9me3, rabbit
	Lamin A, mouse		Lamin A, mouse
3	H1.4, rabbit	7	γH2AX, rabbit
	H1.0, mouse		J2, mouse
4	H1.5, rabbit		
	Lamin A, mouse		

Supplementary Figure Legends

Supplementary Figure S1. β-galactosidase staining of T47D control and senescence induced cells. (**A**) Cells treated with doxorubicin (DR) 0.05 µM, 0.1 µM and 0.5 µM. (**B**) Cells treated with alisertib (AL) 0.5 µM, 1 µM and 3 µM. (**C**) Cells treated with etoposide (ET) 0.1 µM, 0.5 µM and 3 µM. Staining and imaging after two and five days. Red asterisks mark the conditions further used along this study. Related to **Figure 1.**

Supplementary Figure S2. Effects of doxorubicin on the expression of H1 variant genes, senescence markers, ISGs and TEs. T47D-derived cells harboring shRNA for H1.2 (**A**) or H1.4 (**B**) KD (without doxycycline) were treated for five days with doxorubicin (DoxoR) 0.05 µM or left untreated (control), and gene expression was measured by RT-qPCR in triplicate with oligonucleotides specific for $p21^{CIP1/WAF1}$ and $p16^{INK4a}$, six H1 variant genes, transposable elements of five classes , or five ISGs, as in **Figure 1**. GAPDH gene was used for normalization and data expressed as fold-change relative to untreated control (dashed line marks fold-change=1). Statistical differences *±DoxoR* are supported by t-test. (***) p-value≤0.005; (**) p-value≤0.01; (*) p-value≤0.05; (ns/non-significant) p-value>0.05. Related to **Figure 1.**

Supplementary Figure S3. Immunoblotting of H1 variants and H3K9me3 upon senescence induction. (**A**) Immunoblotting of H1 variants H1.1 to H1.5, H1X and the PTM H3K9me3 on seven different membranes, with histone 3 (H3) blotting as loading control and Coomassie staining of the SDS-PAGE gel after protein transfer to the membrane. T47D cells were untreated (C) or treated for 5 days with doxorubicin 0.05µM (DR), alisertib 0.5µM (AL) and etoposide 0.5µM (ET) and total histones extracted. (**B**) Immunoblotting of H1.0 on six of the seven membranes used in (**A**) due to the compatibility of secondary antibodies raised against rabbit (H1 variants) or mouse (H1.0) primary antibodies. Molecular weight marker (M) is shown. Related to **Figure 1D.**

Supplementary Figure S4. Effect of H1 variant depletion and drug-induced senescence on cell viability. T47D cells harboring a doxycycline-inducible lentivirus for the knock-down (KD) of H1.0, H1.2 or H1.4 were treated with doxycycline (±Dox) for 6 days to induce H1 KD and with two different concentrations of doxorubicin (DoxoR C1=0.01 and C2=0.05 µM) for 5 days or left untreated. Cells were analyzed by FACS and gated as live cells in the FSC and SSC scatter plot to distinguish them from debris or enlarged cells. Results are expressed as % of live cells. Related to **Figure 3A**.

Supplementary Figure S5. Effect of H1 variant depletion and doxorubicin treatment on gene expression. T47D cells harboring the inducible system for H1.2 (A), H1.4 (B) or H1.0 (C) KDs were treated ± Dox and ±DoxoR C1 or C2 as in **Figure 3**, after RNA extraction, gene expression was measured by RT-qPCR for the indicated genes. GAPDH gene was used for normalization and data expressed relative to the untreated sample. Representative experiments analyzed in triplicate are shown. Statistical differences ±Dox (H1 KD) or ±DoxoR are supported by t-test. (***) p-value≤0.005; (**) p-value≤0.01; (*) p-value≤0.05; (ns/non-significant) p-value>0.05. Related to **Figure 4**.

Supplementary Figure S6. Intensity profile of TOP2A and DNA staining in WT and H1.4 KD cells treated with doxorubicin. WT and H1.4 KD cells were treated ± Dox and ±DoxoR C2 and submitted to immunofluorescence with antibody recognizing TOP2A and DAPI stained. Plots show the intensity profile of TOP2A (in red) and DNA (in blue) staining along a depicted line used to measure the cell diameter for two representative cells per condition. Related to **Figure 5B-D**.

Supplementary Figure S1

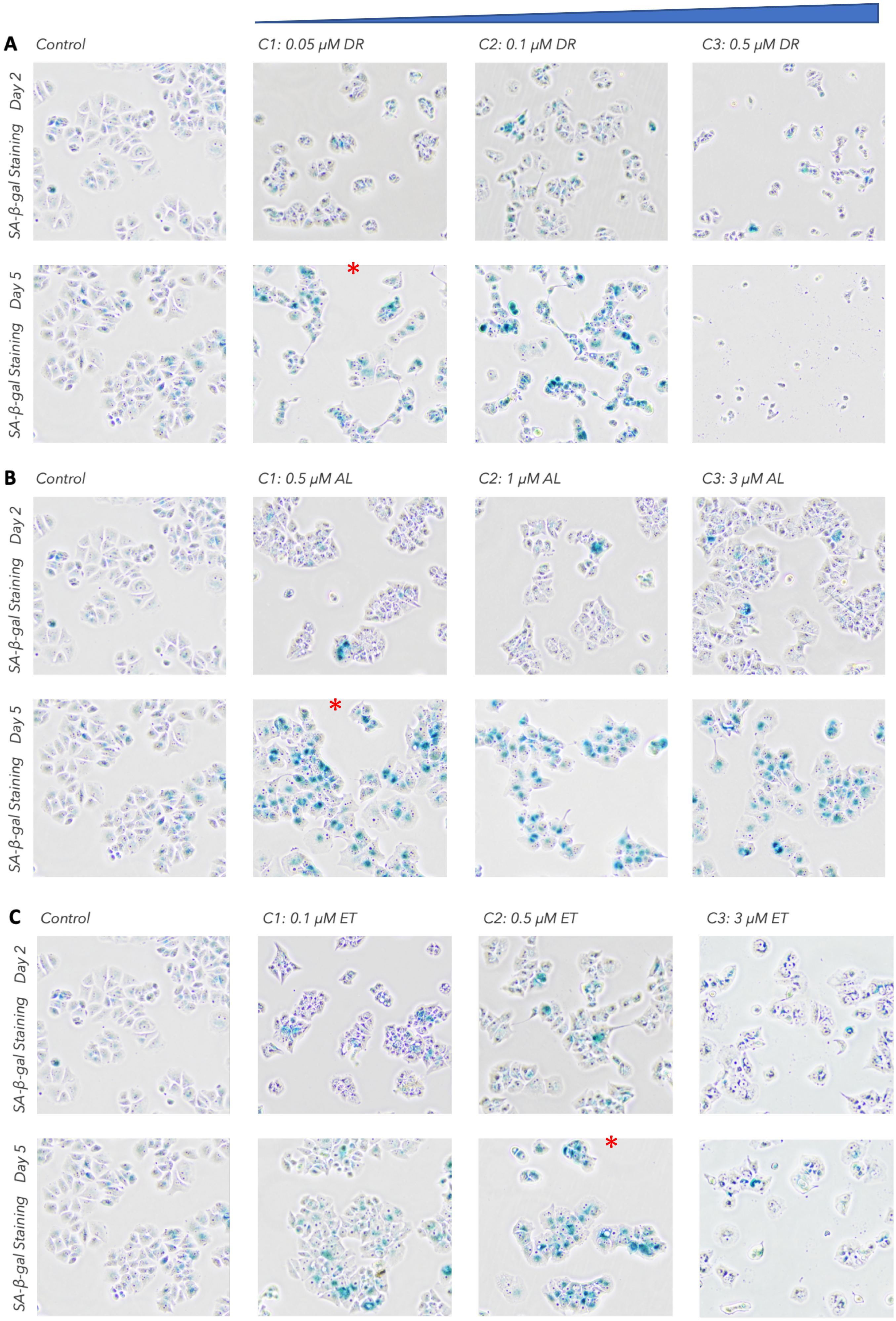

Supplementary Figure S2

A

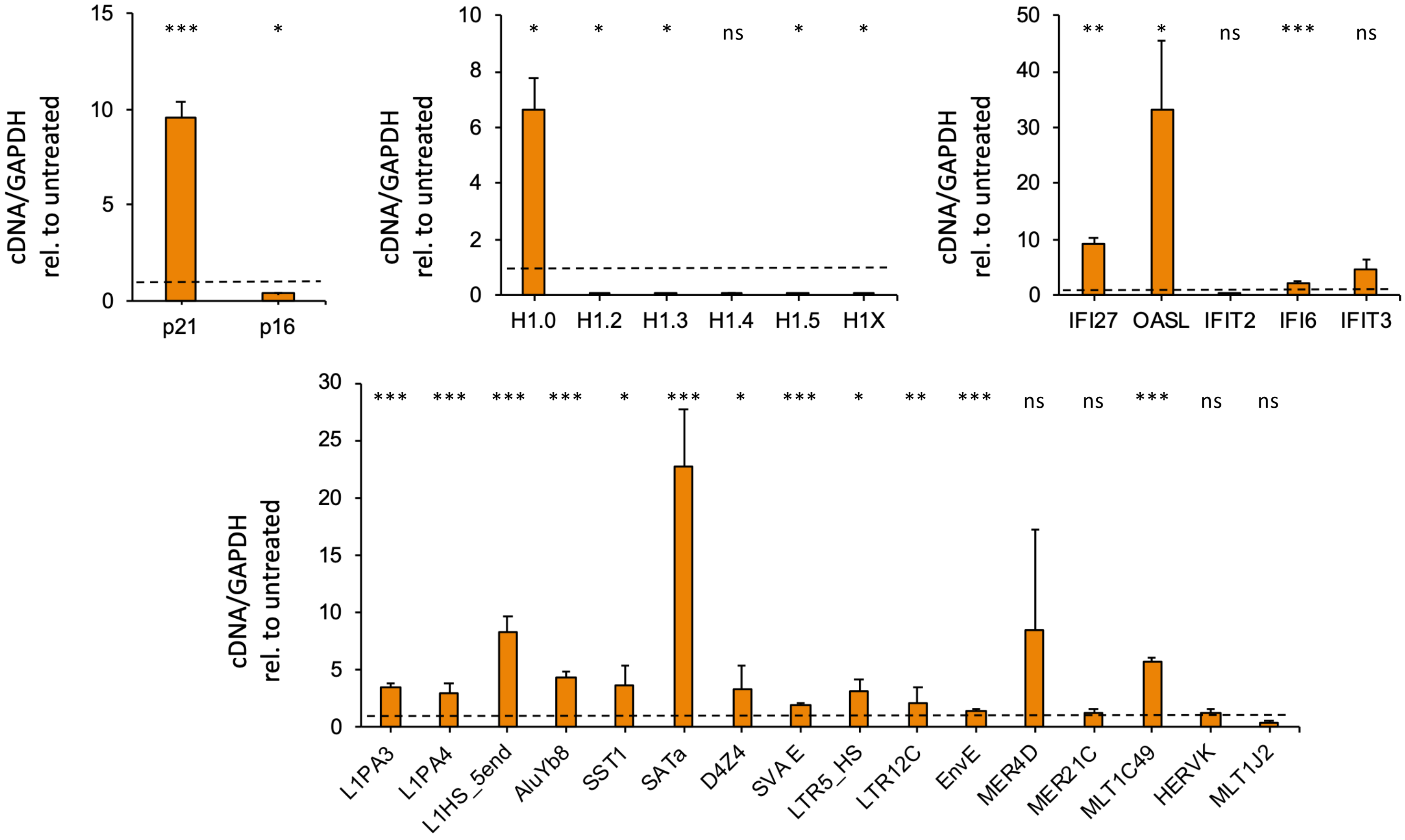

B

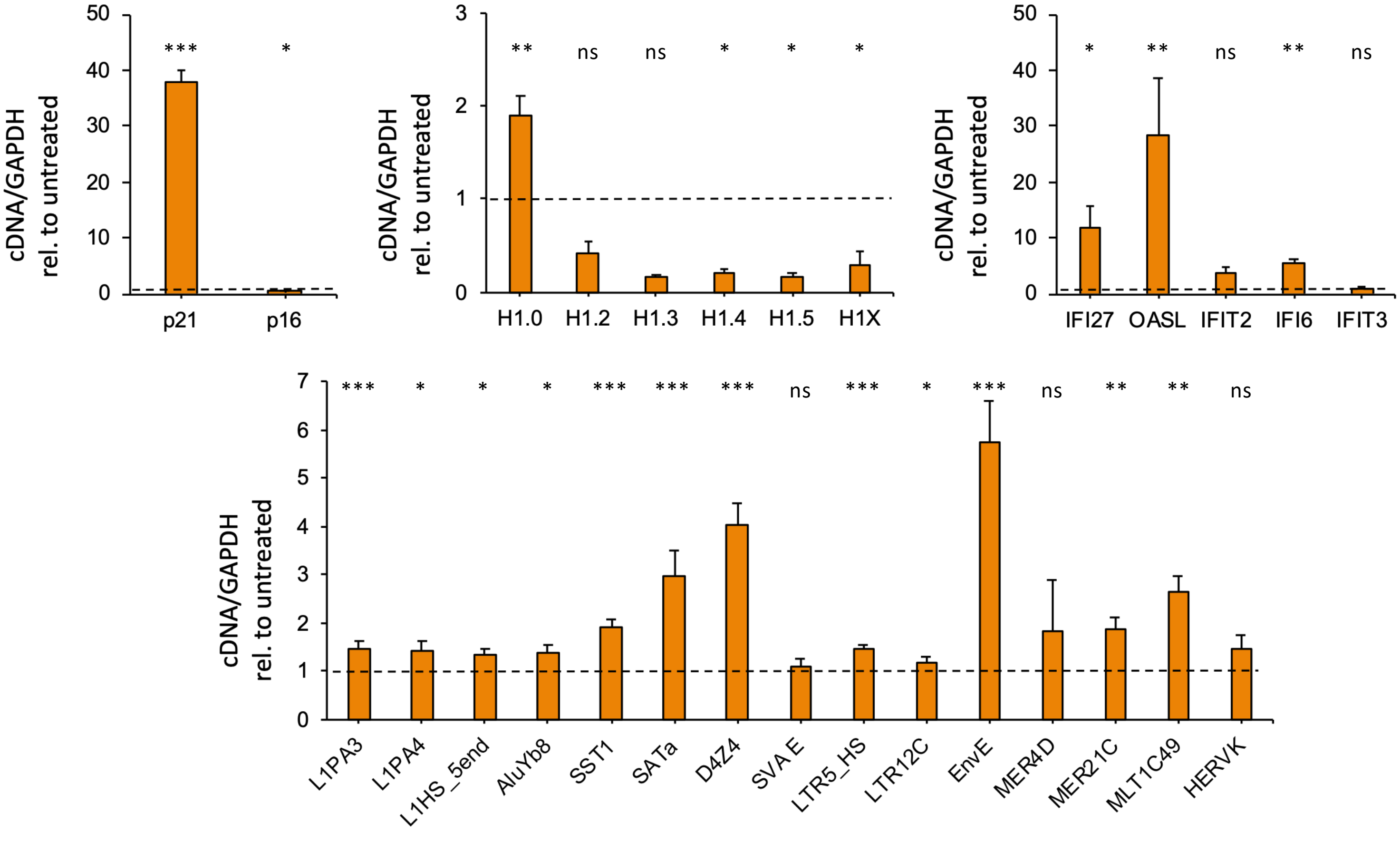

Supplementary Figure S3

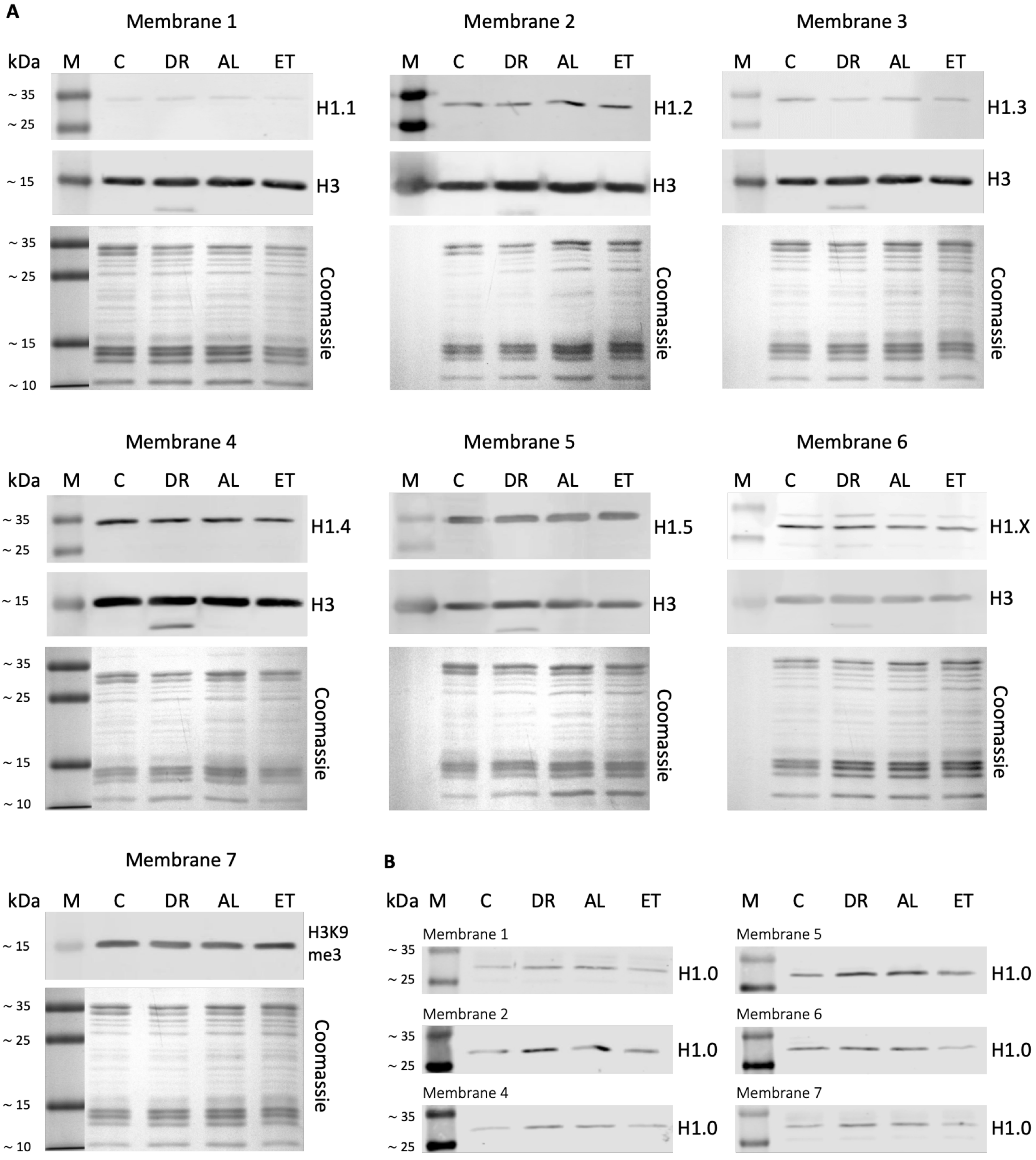

Supplementary Figure S4

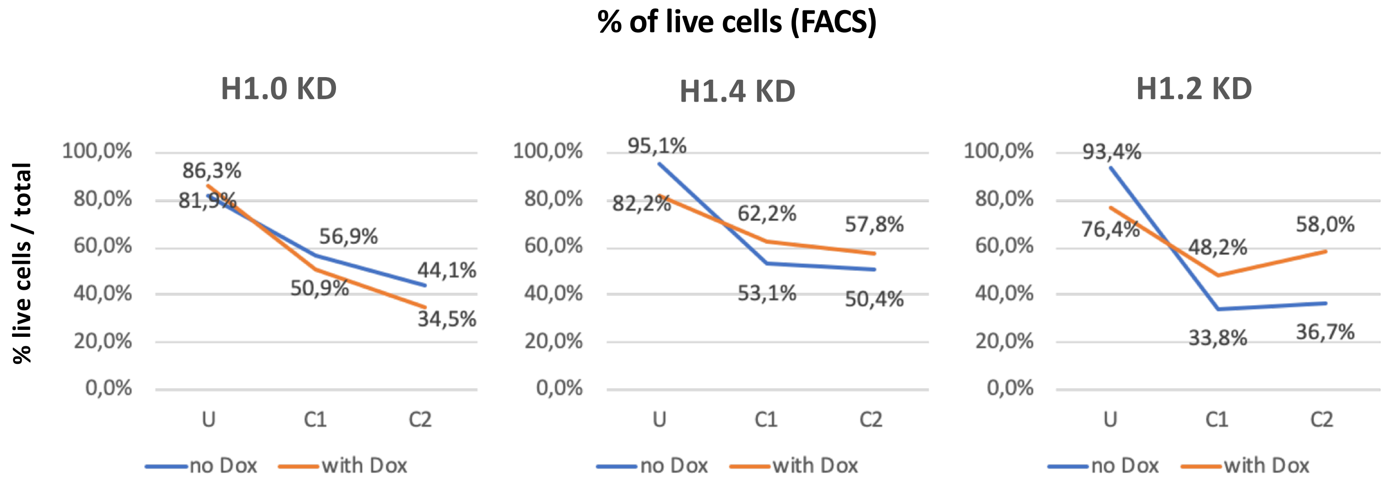

Supplementary Figure S5

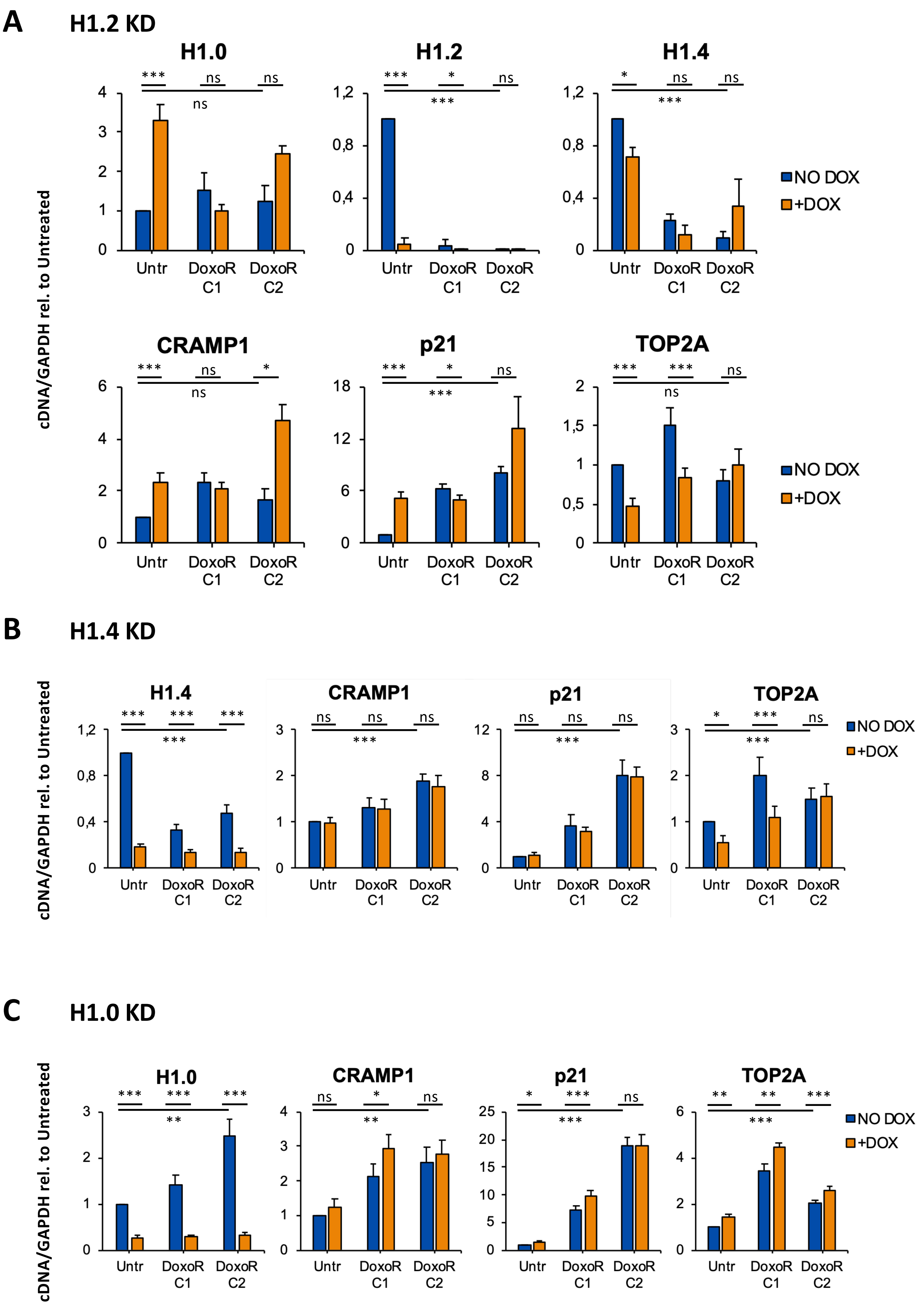

Supplementary Figure S6

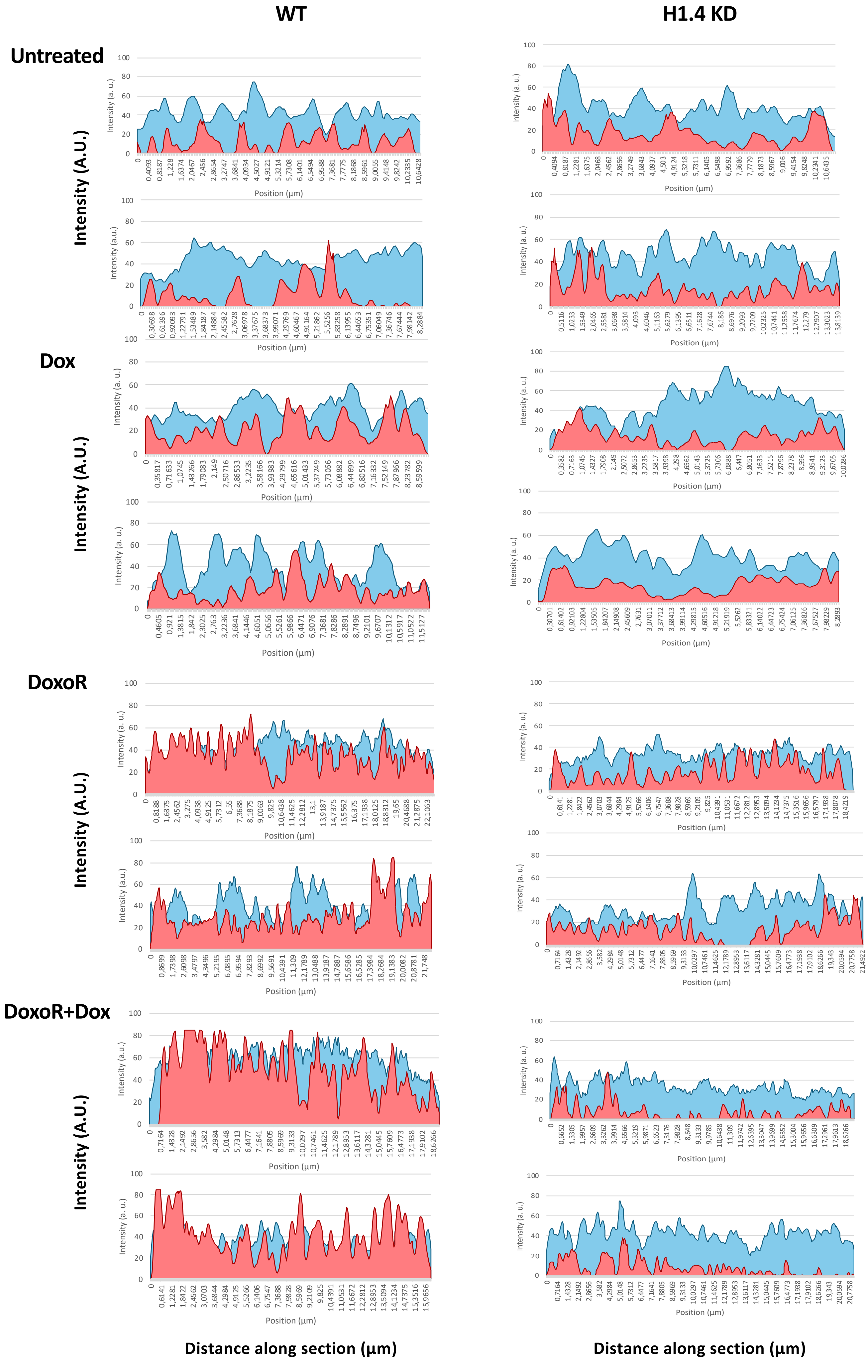

Supplement: Supplementary file 1 [file genes-17-01274-s001.zip › genes-4504615-supplementary.pdf]
